# *In Vivo* Senescence in the Sbds-Deficient Murine Pancreas: Cell-Type Specific Consequences of Translation Insufficiency

**DOI:** 10.1371/journal.pgen.1005288

**Published:** 2015-06-09

**Authors:** Marina E. Tourlakis, Siyi Zhang, Heather L. Ball, Rikesh Gandhi, Hongrui Liu, Jian Zhong, Julie S. Yuan, Cynthia J. Guidos, Peter R. Durie, Johanna M. Rommens

**Affiliations:** 1 Program in Genetics & Genome Biology, Research Institute, The Hospital for Sick Children, Toronto, Canada; 2 Department of Molecular Genetics, University of Toronto, Toronto, Canada; 3 Program in Developmental and Stem Cell Biology, Research Institute, The Hospital for Sick Children, Department of Immunology, University of Toronto, Toronto, Canada; 4 Program in Physiology & Experimental Medicine, Research Institute, Division of Gastroenterology & Nutrition, The Hospital for Sick Children, Department of Paediatrics, University of Toronto, Toronto, Canada; University of California, San Francisco, UNITED STATES

## Abstract

Genetic models of ribosome dysfunction show selective organ failure, highlighting a gap in our understanding of cell-type specific responses to translation insufficiency. Translation defects underlie a growing list of inherited and acquired cancer-predisposition syndromes referred to as ribosomopathies. We sought to identify molecular mechanisms underlying organ failure in a recessive ribosomopathy, with particular emphasis on the pancreas, an organ with a high and reiterative requirement for protein synthesis. Biallelic loss of function mutations in *SBDS* are associated with the ribosomopathy Shwachman-Diamond syndrome, which is typified by pancreatic dysfunction, bone marrow failure, skeletal abnormalities and neurological phenotypes. Targeted disruption of Sbds in the murine pancreas resulted in p53 stabilization early in the postnatal period, specifically in acinar cells. Decreased Myc expression was observed and atrophy of the adult SDS pancreas could be explained by the senescence of acinar cells, characterized by induction of Tgfβ, p15^Ink4b^ and components of the senescence-associated secretory program. This is the first report of senescence, a tumour suppression mechanism, in association with SDS or in response to a ribosomopathy. Genetic ablation of p53 largely resolved digestive enzyme synthesis and acinar compartment hypoplasia, but resulted in decreased cell size, a hallmark of decreased translation capacity. Moreover, p53 ablation resulted in expression of acinar dedifferentiation markers and extensive apoptosis. Our findings indicate a protective role for p53 and senescence in response to Sbds ablation in the pancreas. In contrast to the pancreas, the Tgfβ molecular signature was not detected in fetal bone marrow, liver or brain of mouse models with constitutive Sbds ablation. Nevertheless, as observed with the adult pancreas phenotype, disease phenotypes of embryonic tissues, including marked neuronal cell death due to apoptosis, were determined to be p53-dependent. Our findings therefore point to cell/tissue-specific responses to p53-activation that include distinction between apoptosis and senescence pathways, in the context of translation disruption.

## Author Summary

Growth of all living things relies on protein synthesis. Failure of components of the complex protein synthesis machinery underlies a growing list of inherited and acquired multi—organ syndromes referred to as ribosomopathies. While ribosomes, the critical working components of the protein synthesis machinery, are required in all cell types to translate the genetic code, only certain organs manifest clinical symptoms in ribosomopathies, indicating specific cell-type features of protein synthesis control. Further, many of these diseases result in cancer despite an inherent deficit in growth. Here we report a range of consequences of protein synthesis insufficiency with loss of a broadly expressed ribosome factor, leading to growth impairment and cell cycle arrest at different stages. Apparent induction of p53-dependent cell death and arrest pathways included apoptosis in the fetal brain and senescence in the mature exocrine pancreas. The senescence, considered a tumour suppression mechanism, was accompanied by the expression of biomarkers associated with early stages of malignant transformation. These findings inform how cancer may initiate when growth is compromised and provide new insights into cell-type specific consequences of protein synthesis insufficiency.

## Introduction

The protein translation machinery encompasses interrelated processes of ribosome biogenesis [1] as well as protein synthesis [2]. Mutations in genes that encode components of this machinery are implicated in a growing list of inherited and acquired disorders termed ribosomopathies. All aspects of cell growth require protein synthesis and deficiency in machinery biogenesis or function can be anticipated to have systemic effects with reduced growth caused by translation insufficiency. This was observed in the *Drosophila minutes* that were initially identified by diminutive size, and are now known to possess mutations in ribosome related genes [3]. Nevertheless, ribosomopathies present as clinical syndromes with select organ failure, often including the bone marrow [4,5]. The mechanisms dictating which organs are affected by any given ribosomopathy are unknown. Susceptibility to organ failure may reflect specific cell type expression levels or threshold requirements for translation [6]. Developmental requirements during organ expansion [6,7] and functional requirements during cued response to extrinsic signals may add other levels of complexity.

Most ribosomopathies are cancer predisposition syndromes. They can be associated with increased risk of hematological malignancies, and solid tumours have also been reported [4]. Numerous studies have linked defects in translational control and ribosome gene dosage to aberrant growth [8,9]. However, studies have primarily discussed cancer progression in the context of increased ribosome biogenesis and/or translation. What precipitates malignancies in a growth-disadvantaged context such as that of a ribosomopathy remains poorly understood.

A number of consequences have been noted with loss of the highly conserved ribosome-associated protein SBDS and its orthologs in various model systems with a common thread of deregulated protein synthesis. There are several lines of evidence indicating that SBDS functions in ribosome metabolism [10,11], specifically with eukaryotic initiation factor 6 (EIF6) and elongation factor Tu GTP binding domain containing 1 (EFTuD1) protein [12,13]. EIF6 is required for binding and maturation of the 60S ribosomal subunit [14,15] and has been shown to block ribosome subunit joining for formation of the 80S ribosome [16,17]; hence EIF6 is considered to limit translation initiation [18]. Gain of function mutations in the yeast ortholog of *Eif6* rescued the severe slow-growth phenotype of SBDS-null yeast strains (*sdo1Δ*) and EFTuD1-null yeast strains (*ria1Δ*) [13,19]. The removal of EIF6 from the 60S ribosomal subunit was shown to require the GTPase activity of EFTuD1 [19]. Further, genetic and protein interactions between homologs of EFTuD1 and SBDS have been demonstrated [11,12,20,21]. The current working molecular model is that SBDS acts with EFTuD1 to promote EIF6 removal from the 60S ribosomal subunit [12,22,23].

Shwachman-Diamond syndrome (SDS) is a recessive ribosomopathy caused by biallelic loss-of-function mutations in *SBDS* [24]. SDS is a multisystem disorder presenting typically within the first year of life with failure to thrive, chronic infection and low blood counts [25]. Exocrine pancreatic dysfunction and blood lineage cytopenia (most often neutropenia) are defining features [26]. Other clinical findings include skeletal defects, decreased brain volume and cognitive impairment [27–30]. SDS is associated with high risk of hematological malignancies (up to 30%) [31]; more recently, early onset solid tumours have also been observed, notably including pancreatic carcinoma [32–34]. The exocrine pancreas has amongst the highest requirements for translation in the body as the site of reiterative digestive enzyme production [35]; SDS pancreatic dysfunction is characterized by severe digestive enzyme deficiency [36].

Studies in both patient-derived cell lines [21,37–39] and animal models of SDS [12,13,21,23,40,41] have demonstrated a role for SBDS in ribosome maturation and ribosome subunit joining. Furthermore, decreased global translation was demonstrated in mouse embryonic fibroblasts with disease-associated mutations of *Sbds* [41] and in human embryonic kidney 293 cell lines depleted for SBDS by siRNA [42]. It remains to be determined how SDS-related disruptions in translation manifest as acute dysfunctions in select organs.

Senescence is a permanent cell cycle arrest associated *in vivo* with tumour suppression and aging. In the context of tumours, senescence is considered to act as a rapid response to aberrant growth, particularly downstream of oncogene induction (e.g. RAS activation) [43]. Engagement of tumour suppressors including p53, CDKN2A (p16^INK4A^), pRB [43], TGFβ and CDKN2B (p15^INK4B^) [44,45], can initiate this permanent arrest of the cell cycle that is associated with quiescent cells that secrete inflammatory cytokines (senescence-associated secretory phenotype) and express senescence-associated β–galactosidase activity (SAβG) [43].

Here we sought to identify *in vivo* mechanisms underlying pancreas dysfunction, in comparison to other organs, in SDS. We used constitutive and targeted mouse models to establish the timing and type of organ responses to *Sbds* mutation. Specifically, we show the dependence of many responses on p53 and that SDS-related translation insufficiency induces a senescent cell cycle arrest through the induction of Tgfβ and p15^Ink4b^ in the murine exocrine pancreas. Our study provides new insights into organ selectivity and tumorigenic potential in ribosomopathies.

## Results

### Loss of Sbds function results in growth and morphological defects

**Fig 1:**
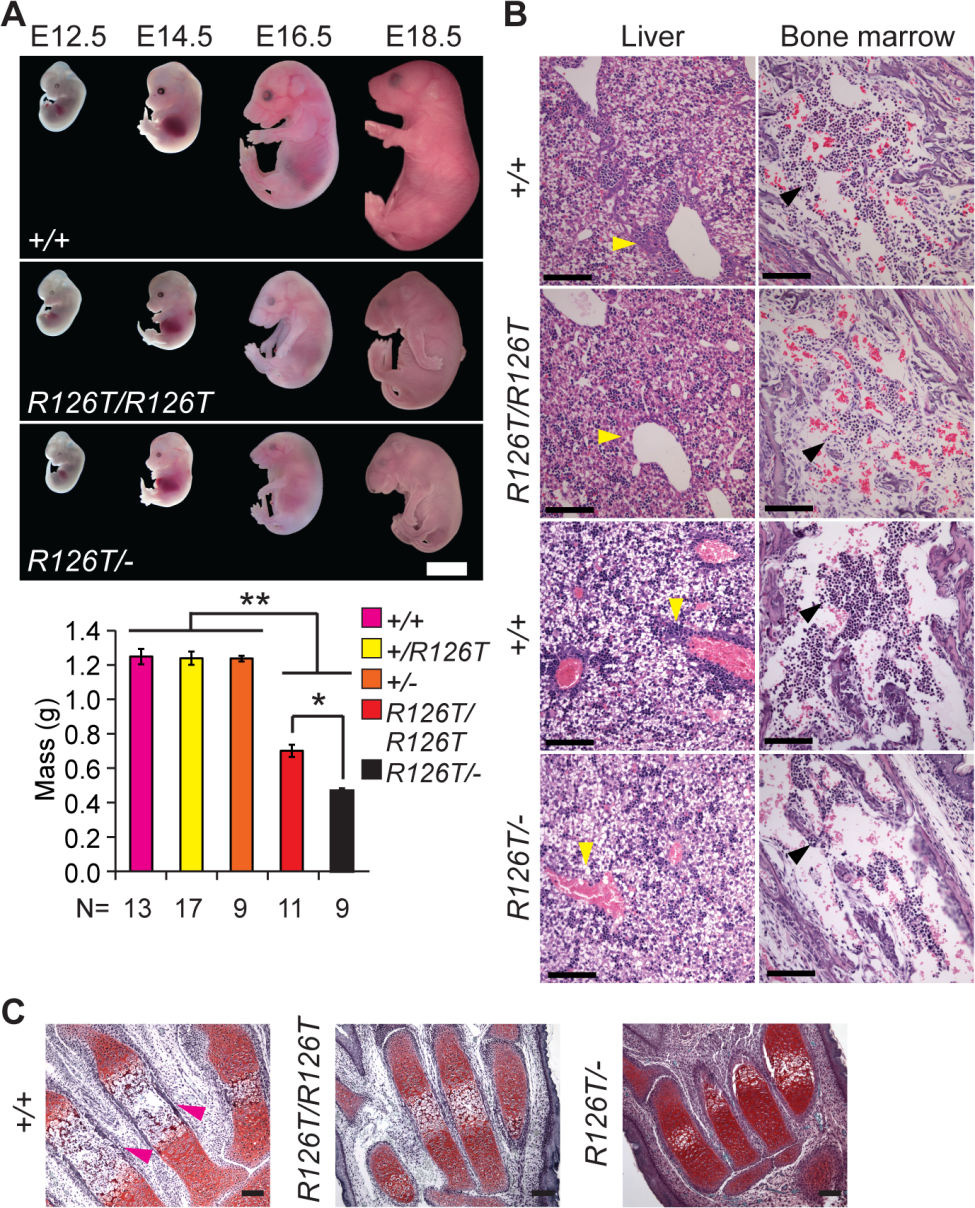
Sbds mutants display ribosomopathy and SDS phenotypes. **A,** Embryos with biallelic mutations in *Sbds* have decreased mass compared with littermate controls, **P<4X10^-6^. *Sbds* ^*R126T/–*^embryos are smaller than *Sbds* ^*R126T/R126T*^ embryos, *P = 1.9X10^-4^ (Wilcoxon Rank Sum Test; Kruskal-Wallis *P* = 3.0X10^-8^). Error bars represent ±SEM. Scale bar represents 5 mm (upper panel). **B,** Decreased granulocytes (dark purple, H&E, E18.5) in liver (cell cluster examples are indicated with yellow arrowheads) and bone marrow (black arrowheads) with loss of *Sbds*; N = 3 (*Sbds* ^*R126T/R126T*^) and 4 (*Sbds* ^*R126T/–*^). Scale bars represent 100 μm. **C,** Decreased bone ossification was observed in transverse metacarpal sections of mutants (corresponding regions of littermate controls that maintain red Safranin O staining in mutants are highlighted with magenta arrowheads, E18.5). Scale bars represent 100 μm.

Mouse models with disease-associated missense (*R126T*) and null (*–*) alleles, *Sbds* ^*R126T/R126T*^ and *Sbds* ^*R126T/–*^, displayed severe growth impairment and did not survive birth (S1 Table; [46]). Models demonstrated complete penetrance and consistent genotype-phenotype correlations, with more severe and earlier onset of disease phenotypes in the *Sbds* ^*R126T/–*^embryos compared to *Sbds* ^*R126T/R126T*^ embryos (Fig 1). Heterozygous carriers of either the *Sbds* ^*−*^or *Sbds* ^*R126T*^ alleles were indistinguishable from wildtype, consistent with a recessive mode of inheritance for SDS. Embryos were visibly smaller by two weeks gestation and at E18.5 were, on average, 38% (*Sbds* ^*R126T/–*^) and 56% (*Sbds* ^*R126T/R126T*^) of age-matched controls by mass (Figs 1A and S1A). Embryo length was also reduced (S1C Fig).

SDS mouse models recapitulated several features observed in human disease. Mutations in *Sbds* are associated with defects in hematopoiesis [47]. In the fetal period, the liver is the primary site of definitive hematopoiesis. In the *Sbds* ^*R126T/R126T*^ model near birth (E18.5), histopathology indicated decreased granulocytes in portal areas of the liver as well as pronounced bone marrow hypocellularity, with increased severity in the *Sbds* ^*R126T/–*^model (Fig 1B).

SDS is also characterized by decreased ossification and delayed bone growth [27,48]. No gross skeletal defects were apparent in the constitutive models [41]; however, ossification was reduced in the metacarpals at late gestation (Fig 1C). In severe cases, asphyxiating thoracic dystrophy has been observed in SDS [49,50], presumably due in part to the skeletal dystrophy. Beyond this, lung pathology has not been specifically reported in SDS patients. We did observe a severe decrease in saccule expansion in the late fetal lung, despite presence of lung developmental stage biomarkers (S2A Fig).

A defining morphological feature of SDS is a small, fat-replaced pancreas [25,27,30]; we previously showed that pancreatic growth impairment, dysfunction and lipomatosis manifest only in the postnatal period [51].

### Proliferation defects and cell death in absence of Sbds function *in utero*

Translation insufficiency impacts all tissues, and all ribosomopathies are associated with poor overall growth. To further investigate the observed decreases in granulocytes in the liver and hypoplasia of the bone marrow compartment we assessed the abundance of hematopoietic progenitors in the fetal liver of the SDS mouse models. Primary myocult cultures derived from E14.5 fetal livers revealed markedly decreased levels of all myeloid lineage progenitors in both the *Sbds* ^*R126T/R126T*^ and *Sbds* ^*R126T/–*^models (S3 Fig). Decreased levels of granulocytes in the *Sbds* ^*R126T/–*^model were also determined by flow cytometry of fetal liver cells (E16.5, S4A Fig). Unlike ribosome deficiency models with dominant inheritance [52–54], erythrocyte levels prior to birth (E18.5) were normal in both mouse models (S4B Fig), consistent with observations in SDS patients [31].

**Fig 2:**
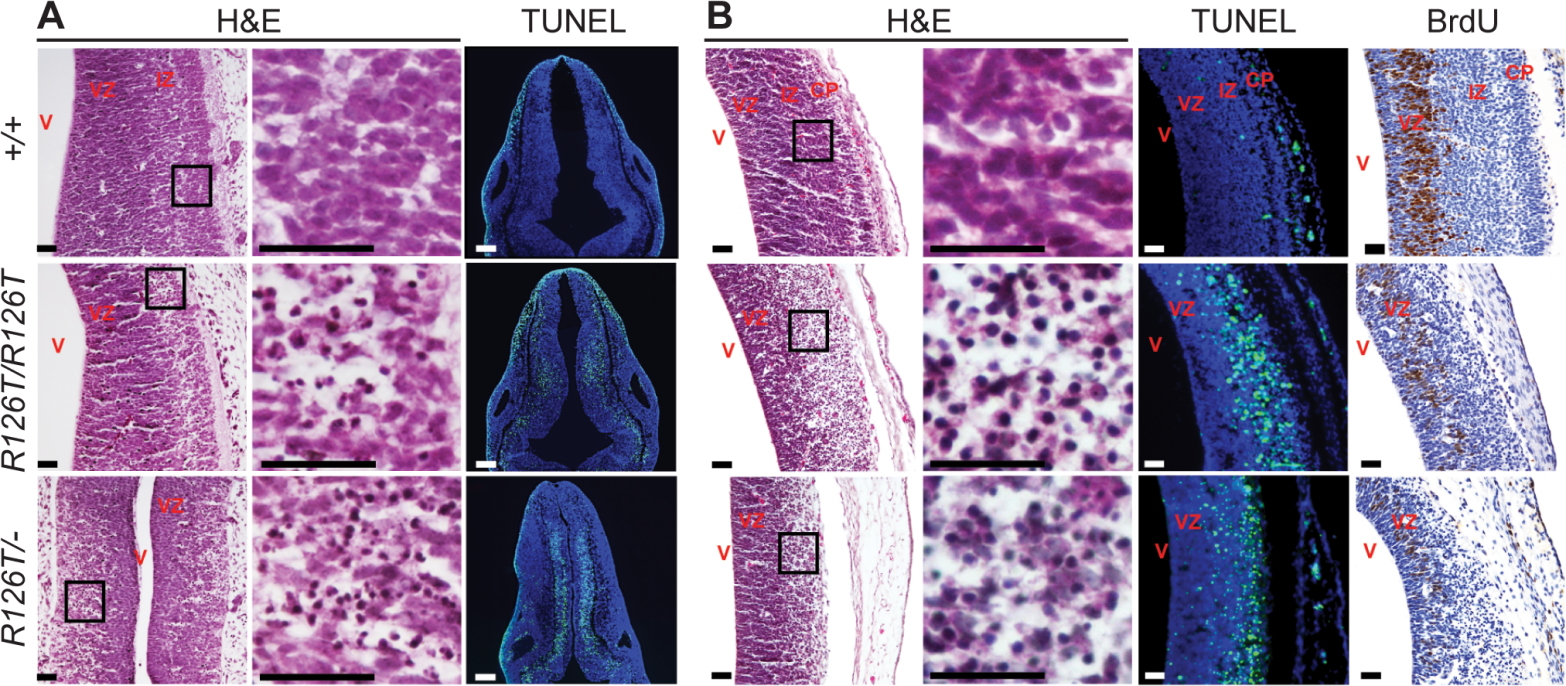
SDS brain is apoptotic. Histochemistry of transverse rhombencephalon (E11.5; **A**) and telencephalon (E14.5; **B**) brain sections indicate hypocellularity and neuronal cell death (green in TUNEL panels) in post-mitotic regions of *Sbds* ^*R126T/R126T*^ mice with earlier onset in the *Sbds* ^*R126T/–*^mice (compare TUNEL panels in **A** and **B**). Bromodeoxyuridine labeling (brown in BrdU panels) highlighted reduced proliferation of neural progenitors. V, lateral ventricle; VZ, ventricular zone; IZ, intermediate zone; CP, cortical plate. Scale bars represent 25 μm.

In contrast to other organs, *Sbds* mutations resulted in severe proliferation defects with pyknotic nuclei and apoptosis (detected by TUNEL staining) in the developing brain by E11.5 in both *Sbds* ^*R126T/–*^and *Sbds* ^*R126T/R126T*^ models (Fig 2A). At E14.5, TUNEL staining was very prominent in the intermediate zone and bromodeoxyuridine labeling further indicated poor growth of neuronal progenitors in the ventricular zone of the developing cortices (Fig 2B). By E18.5, the brain showed multifocal lesions of necrotic neurons (S5 Fig). We did not observe an increase in TUNEL staining in other tissues at E18.5, including the liver and bone marrow, beyond what was observed in controls (S6 Fig).

### Loss of Sbds results in senescence in the postnatal pancreas

As mentioned above, the constitutive SDS models did not survive birth. Using a conditional knockout allele (*CKO*) in conjunction with a pancreas-specific Cre driver (*Ptf1a* ^*Cre*^) to circumvent lethality, we previously showed that biallelic loss-of-function mutations in *Sbds* result in a very small pancreas (53% of controls, relative to body mass [51]) with severe atrophy of the acinar component of the adult pancreas. This phenotype included a dramatic depletion of zymogen granules, the specialized vesicles that house digestive enzymes in pancreatic acinar cells. Furthermore, in contrast to the developing brain, poor pancreatic growth was not explained by apoptosis [51].

**Fig 3:**
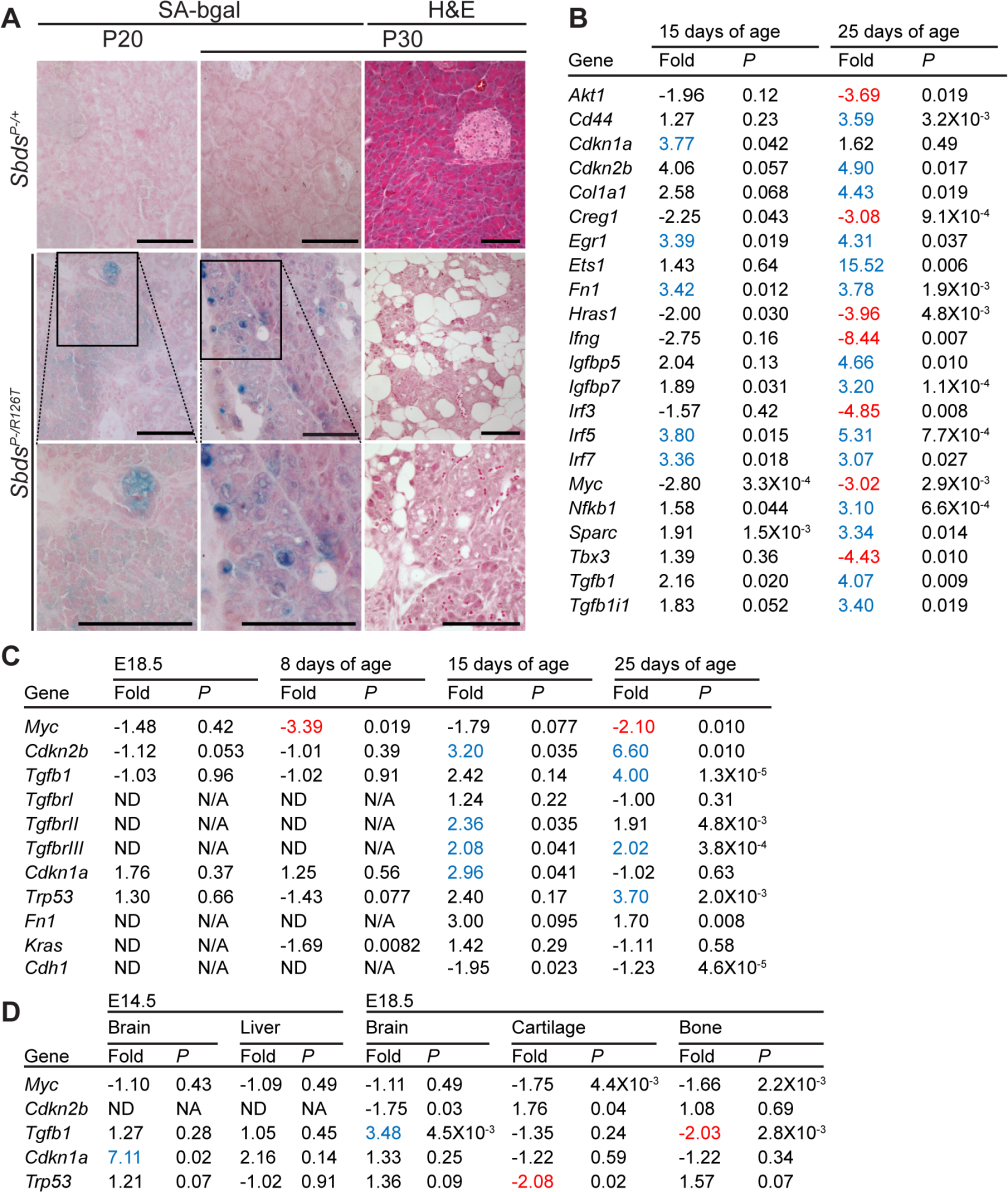
Senescence-associated markers in the SDS pancreas. **A,** Senescence-associated β-galactosidase activity (SA-bgal, bright blue) was detected in acini of the SDS pancreas at 30 days of age (N = 5). *Sbds* ^*P–/+*^ and *Sbds* ^*P–/R126T*^ are shorthand for *Sbds* ^*CKO/+*^; *Ptf1a* ^*Cre/+*^ and *Sbds* ^*CKO/R126T*^; *Ptf1a* ^*Cre/+*^, respectively [51]. Scale bars represent 100 μm. **B,** 84 cellular-senescence associated genes were assayed using the SABiosciences Cellular Senescence RT^2^ Profiler PCR Array (QIAGEN) with total RNA isolated from pancreata of mice at 15 and 25 days of age. Table lists transcripts that showed statistically significant changes relative to control genes at at least one of the two assayed time points (see also S2 Table). Fold change: *Sbds* ^*P-/R126T*^/*Sbds* ^*P-/+*^. Criteria for significance (as per supplier’s instructions): ≥3 fold difference with a *P*-value of <0.05, N = 3 at each time point. **C** and **D,** Quantitative transcript analysis. In **C**, fold change: *Sbds* ^*P-/R126T*^/*Sbds* ^*P-/+*^; N = 4 at each time point, except at E18.5 where N = 3. Criteria for significance: ≥2 fold change, *P*<0.05. E18.5 pancreas expression is relative to Tbp; P8-P25 expression is relative to Gapdh. In **D**, fold change: *Sbds* ^*R126T/R126T*^/*Sbds* ^*R126T/+*^; N = 4. Criteria for significance: ≥2 fold change, *P*<0.05. Brain and liver expression is relative to Actb; bone expression is relative to Tbp. All *P*-values calculated using unpaired, two-tailed T-tests. Red indicates down-regulation, blue indicates up-regulation. Abbreviations in **B**: *Akt1*: *Thymoma viral proto-oncogene 1; Cd44*: *CD44 antigen; Cdkn1a*: *Cyclin-dependent kinase inhibitor 1A; Ckdn2b*: *Cyclin-dependent kinase inhibitor 2B; Col1a1*: *Collagen*, *type I*, *alpha 1; Creg1*: *Cellular repressor of E1A-stimulated genes 1; Egr1*: *Early growth response 1; Ets1*: *E26 avian leukemia oncogene 1*, *5’ domain; Fn1*: *Fibronectin 1; Hras1*: *Harvey rat sarcoma virus oncogene 1; Ifng*: *Interferon gamma; Igfbp5*: *Insulin-like growth factor binding protein 5; Igfbp7*: *Insulin-like growth factor binding protein 7; Interferon regulatory factor 3; Irf5*: *Interferon regulatory factor 5; Irf7*: *Interferon regulatory factor 7; Myc*: *Myelocytomatosis oncogene; Nfkb1*: *Nuclear factor of kappa light polypeptide gene enhancer in B-cells 1*, *p105; Sparc*: *Secreted acidic cysteine rich glycoprotein (osteonectin; Tbx3*: *T-box-3; Tgfb1*: *Transforming growth factor*, *beta 1; Tgfb1i1*: *Transforming growth factor beta 1 induced transcript 1*.

Given the loss of zymogen granules and acinar cell hypoplasia with a persistent absence of cell death markers, we considered that a senescent cell-cycle arrest might explain atrophy of the SDS pancreas. Several acinar cells of the SDS pancreas were positive for SAβG activity by 20 days of age, becoming more prominent by 30 days of age (Fig 3A). With this evidence of senescence, we next investigated the nature of this response by performing transcript analyses with reverse-transcriptase real-time quantitative PCR of a curated cellular senescence panel of genes. Pancreas samples from littermate control-mutant pairs were compared at two time points prior to the pronounced SAβG activity (Fig 3B, S2 Table). SDS pancreas transcripts showed a suite of changes that were, consistent with the literature, indicative of a senescent-associated cell cycle arrest and secretory program [55–57]. We then further investigated targets of the p53/p21^Cip1^ and Tgfβ/p15^Ink4b^ networks with additional samples and time points (Fig 3C). We detected markedly increased expression of p15^Ink4b^ (Cdkn2b) along with Tgfβ together with low Myc expression at 15 and 25 days of age (Fig 3C). Increased expression of p21^Cip^ (Cdkn1a) occurred at the early time point of 15 days. Consistent with low Myc levels being permissive for p15^Ink4b^ induction by Tgfβ [58], decreases in Myc transcript levels were noted already at one-week of age, preceding increases in Tgfβ and p15^Ink4b^ (Fig 3C). An increase in p53 transcript expression (3.70 fold, relative to controls), a known mediator of the senescence response [43], also coincided with the onset of SAβG activity (Fig 3C).

**Fig 4:**
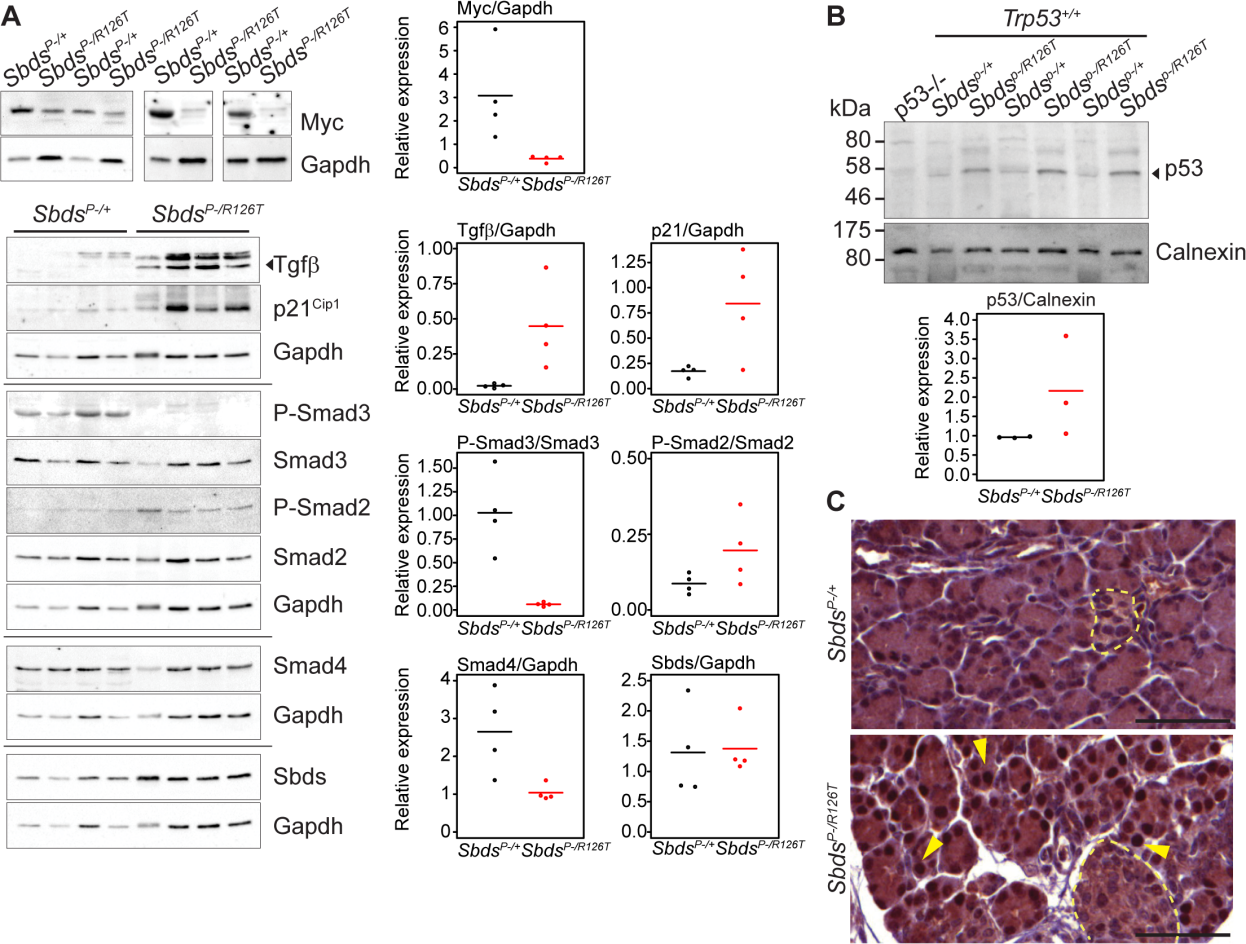
Tgfβ and p53 response in the SDS pancreas. **A,** Steady state protein levels paralleled observed transcript changes with decreased Myc and increased p21^Cip^ and Tgfβ expression, along with changes in Smad2 and Smad3 phosphorylation status in mutants. Representative immunoblots of lysates from four littermate pairs at 3 weeks of age are shown. Associated densitometry is shown in right graphs, with Myc, Tgfβ, and p21 relative to Gapdh expression, and phosphorylated-Smad2 and Smad3 relative to total Smad2 and Smad3, respectively. Horizontal lines in scatter plots indicate mean values. **B,** Representative immunoblot indicates stabilization of p53 protein in the SDS pancreas at 3 weeks of age with associated densitometry (expression relative to calnexin) below. Horizontal lines indicate mean values. **C,** Immunohistochemistry indicated p53 stabilization as early as 15 days of age (sections shown are of littermates). Yellow arrows highlight examples of positive nuclei. p53 staining was observed specifically in nuclei of acinar cells of the SDS pancreas model (islets denoted with pale yellow dashed outlines). Scale bars represent 50 μm.

Protein expression analyses of control-mutant littermate pairs from several litters paralleled the transcript changes in the *Sbds* ^*P–/R126T*^ pancreas with changes in Myc and Tgfβ signalling (Fig 4A). Steady-state protein levels of Myc and Tgfβ were consistently reduced and higher in mutants, respectively (Fig 4A). Tgfβ signalling is propagated by phosphorylation of the Smad proteins by Tgfβ receptors [59]. We noted less Smad3 phosphorylation, but more Smad2 phosphorylation in mutants than in controls (Fig 4A). We also observed increased transcript levels for Tgfβ receptors, TgfbrII and TgfbrIII, which can be upregulated during increased Tgfβ signalling [60] (Fig 3C).

Expression of several factors implicated in the senescence-associated secretory phenotype [55,56,61] beyond Tgfβ, were also elevated. These included extracellular matrix proteins fibronectin (Fn1), osteonectin (Sparc) and collagen (Colla1) as well as innate immunity genes (e.g. Irf5, Irf7 and Nfkb1) and insulin growth factor binding proteins (Igfbp5 and Igfbp7) (Fig 3B; S2 Table), consistent with a senescence program.

Notably, indicators of replicative- and oxidative stress-induced senescence (e.g. Sod1 and Akt1, respectively [43]) were not elevated (S2 Table). Further, that expression of proto-oncogenes Akt1, Hras and Kras as well as Myc trended downwards or were reduced refuted an oncogene-induced senescence response (Fig 3B; S2 Table).

Tgfβ is a known driver of epithelial to mesenchymal transition [59] so we also considered that this process may be occurring in the SDS pancreas. We did observe indicators of dedifferentiation in the mature SDS pancreas (see below); however E-cadherin (Cdh1) transcript levels were not significantly reduced at young ages (Fig 3C).

To determine if the molecular signature of the pancreas senescence represented a common response to *Sbds*-ablation, we investigated whether cyclin inhibitors, Tgfβ, and Myc transcript level changes were evident in tissues of the constitutive SDS model (*Sbds* ^*R126T/R126T*^). A marked increase in p21^Cip^ transcript levels in the brain at E14.5 was observed when apoptosis was detected (Fig 3D). At this same early time point, Tgfβ expression was not altered in either mutant fetal brain or liver even though both organs manifested phenotypes (Fig 3D). Tgfβ expression was low in bone (Fig 3D) and unchanged in lung at E18.5 (S2B Fig). By E18.5, Tgfβ expression was elevated in the SDS mouse brain (Fig 3D), likely a late response to brain damage [62]. No changes in p15^Ink4b^ or Myc expression levels were observed in fetal liver, lung, bone or cartilage tissues (Fig 3D; S2B Fig). These findings are consistent with Tgfβ/p15^Ink4b^-mediated senescence being a specific response of the pancreas to Sbds deficiency.

### Growth impairment and senescence in the SDS pancreas are p53-dependent phenotypes

p53 is a known driver of senescent cell cycle arrest [57,63] and increased levels of p53 have been reported in SDS patients [64]. Moreover, studies of ribosomal gene haploinsufficiency have implicated p53 as a key factor in response to ribosome dysfunction [54,65]. We observed increased steady-state levels of p53 protein in the SDS mouse pancreas by immunoblotting (3 weeks of age, Fig 4B). Immunohistochemistry for p53 (15 days of age, prior to the detection of SAβG staining, Fig 4C) specifically highlighted nuclei of acinar cells, but not islet cells (Fig 4C). To determine if the senescence in the SDS pancreas is p53-dependent we bred the SDS pancreas model to a *Trp53* ^*–/–*^mouse.

**Fig 5:**
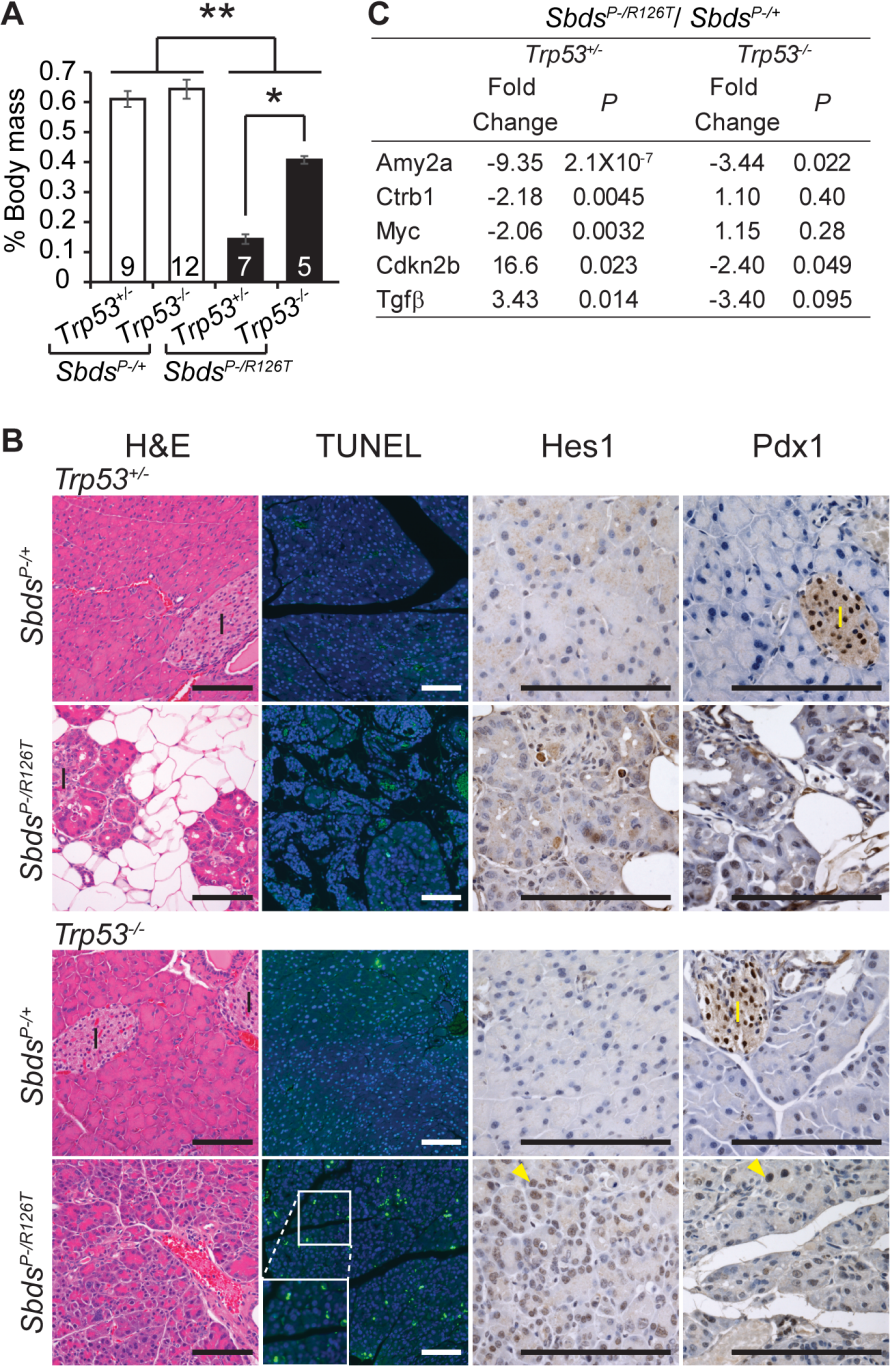
Atrophic SDS pancreas phenotype is p53-dependent. **A,** Improved mass of SDS pancreas with loss of p53 (*Sbds* ^*P–/R126T*^; *Trp53* ^*–/–*^; 30 days of age); *P = 0.0025, **P<4X10^-5^, Wilcoxon Rank Sum Test (Kruskal-Wallis Test *P* = 3.2X10^-5^). Inset numbers = N. Error bars represent ±SEM. **B,** Resolution of atrophy and hypocellularity as well as fat infiltration with loss of p53 in SDS pancreas at 60 days of age (H&E). However, multiple apoptotic acinar cells (TUNEL) per field of view and increased expression of dedifferentiation markers in acini (examples of Hes1 and Pdx1 positive cells, yellow arrowheads) highlighted dysplasia. I: islets. Scale bar represents 100 μm. **C,** Improvement in digestive enzyme expression and abrogation of senescence-related changes in Tgfβ/p15^Ink4b^ and Myc expression in double mutants at 25 days of age. Fold changes correspond to the comparison of *Sbds* ^*P–/R126T*^;*Trp5* ^*+/–*^to *Sbds* ^*P–/+*^;*Trp53* ^*+/–*^or *Sbds* ^*P–/R126T*^;*Trp53* ^*–/–*^to *Sbds* ^*P–/+*^;*Trp53* ^*–/–*^transcript levels. *P*-values calculated using unpaired, two-sided T-tests.

Complete genetic ablation of p53 alleviated the phenotypes of the SDS pancreas. *Sbds* ^*P–/R126T*^;*Trp53* ^*–/–*^animals demonstrated a notable improvement in pancreas mass as compared with *Sbds* ^*P–/R126T*^;*Trp53* ^*+/–*^animals (Fig 5A). Growth improvement was also evident at the histological level as acinar hypoplasia and fat infiltration did not occur in *Sbds/Trp53* double mutants in direct contrast to single *Sbds* mutants (Fig 5B). The molecular signature associated with senescence in the SDS model pancreas was no longer detected; specifically Tgfβ, and p15^Ink4b^ transcripts were not elevated and Myc transcript levels were not decreased (Fig 5C) at 25 days of age. Further, elevated SAβG activity was not detected at 32 days of age (S7 Fig).

By one month of age, the architecture of the acinar epithelium in *Sbds/Trp53* double deficient pancreata appeared disordered with many apoptotic cells evident by two months of age (Fig 5B). The morphology was consistent with early stages of acinar-ductal metaplasia. By 60 days of age, we had already noted that some acinar cells in the SDS model pancreas were positive for transcription factors Hes1 and Pdx1, both of which are associated with dedifferentiation (Fig 5B) [66]. With complete ablation of p53, staining of acinar cells with these dedifferentiation markers became widespread (Fig 5B). In contrast, we did not detect changes in islet structure, nor did islets contain apoptotic cells, consistent with our previous observation that mutations in *Sbds* specifically impact the acinar compartment of the pancreas [51].

### *p53* ^*–/–*^genetic background unmasks underlying translation-insufficiency of *Sbds* ^*P–/R126T*^ pancreas

**Fig 6:**
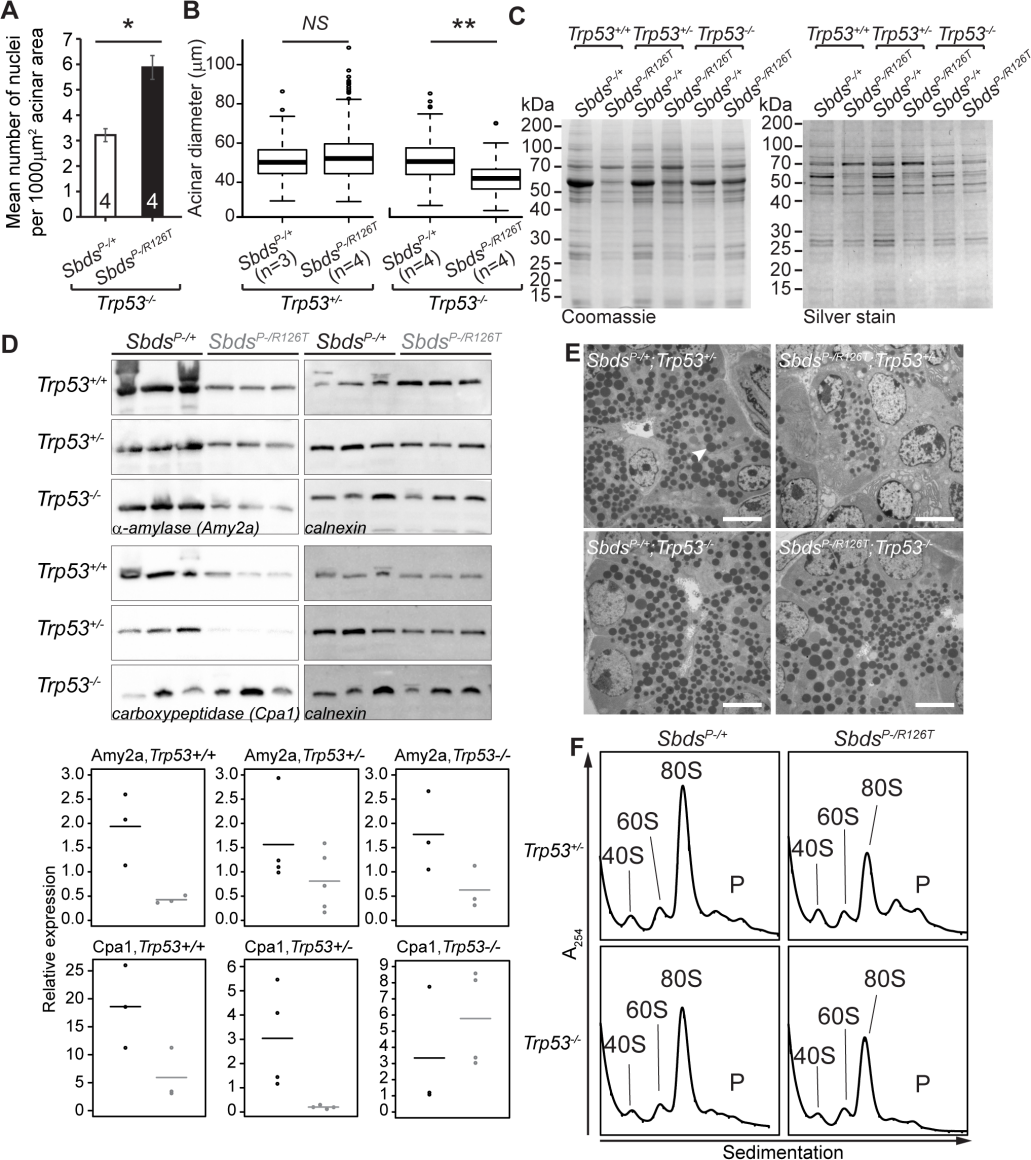
SDS pancreas senescence is downstream of p53-dependent changes in protein synthesis. **A,** Increased nuclei per acinar area (*P = 0.029, Wilcoxon Rank Sum Test) and **B,** decreased mean acinus diameter (**P = 0.0073 and *NS* = not significant, *P* = 0.142; unpaired, two-sided T-test) in *Sbds* ^*P–/R126T*^; *Trp53* ^*–/–*^pancreas at 30 days of age. Error bars represent ±SEM; in boxplots, whiskers represent extreme values; circles, outliers. **C,** Coomassie and silver staining of pancreas lysate SDS-PAGE illustrated p53-dependent altered protein expression in the SDS pancreas (20 days of age, 6 μg total protein loaded). **D,** Immunoblotting confirmed that digestive enzymes were reduced in expression in the SDS pancreas, with expression of protease carboxypeptidase (Cpa1) increasing in a *Trp53* ^*–/–*^genetic background (3 weeks of age, 25 μg total protein loaded). Representative blots are shown. Associated densitometry, with expression relative to Gapdh, is shown in lower panels, *Sbds* ^*P–/+*^ black, *Sbds* ^*P–/R126T*^ grey, horizontal lines indicate mean values. **E,** Restoration of zymogen granules (example, white arrowhead) with loss of p53 was observed by one week of age (electron micrographs). Scale bar represents 5 μm. **F,** Representative polysome traces illustrate restoration of 80S peak in mutants to levels similar to those of controls with loss of p53 (20 days of age, 79 μg RNA loaded, N = 4 (*Trp53* ^*+/–*^) and 3(*Trp53* ^*–/–*^)). P: polysomes.

The absence of p53 further revealed translation-insufficiency as a consequence of Sbds loss-of-function. A long established feature of ribosomal deficiency includes small cell size [7], a phenotype noted for the acinar cells of the *Sbds/Trp53*-double deficient pancreata. Quantification of micrographs of doubly deficient pancreas tissue revealed a nuclei count increase per acinar area compared to *Trp53* ^*–/–*^controls (with Sbds), indicating more cells per area (and hence a decreased cell size; Fig 6A). Correspondingly, a smaller mean acinus diameter was also evident in the double mutant micrographs (Fig 6B).

Despite this indication of ribosomal deficiency, *Sbds/Trp53* double mutants demonstrated a substantial rescue of digestive enzyme expression and zymogen granule abundance. In fact, SDS pancreas lysates showed qualitatively different protein expression patterns that became similar to controls when p53 was absent (Fig 6C). Although amylase expression remained low, increases in protease (carboxypeptidase) expression at three weeks (Fig 6D) as well as restoration of zymogen granules by one week (Fig 6E) in the absence of p53 supported improvement in exocrine function.

We previously suggested that loss of Sbds results in a moderate decrease in 80S monosome peak levels compared to littermate controls [51]. Corresponding increases in free ribosomal subunit levels were not apparent as would be expected if ribosome production was maintained, whereas quantification of the 80S monosome peak levels in the polysome profiles of mutant pancreata showed that the modest decrease normalized to that of controls with ablation of p53 (Figs 6F and S8).

### p53 activation and tissue specific outcomes

**Fig 7:**
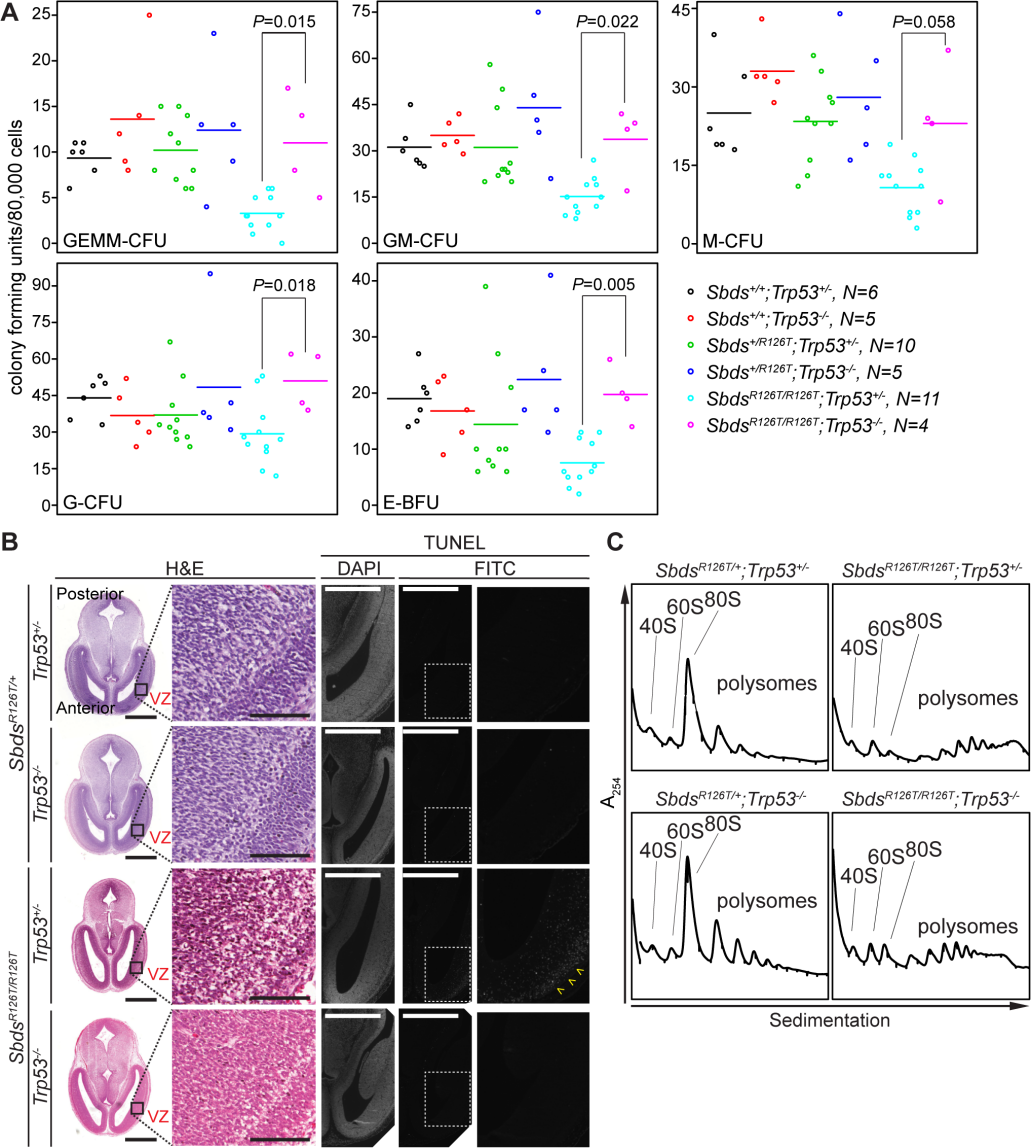
Hematopoietic and neuronal SDS phenotypes are p53-dependent. **A,** Restored hematopoietic progenitor colony forming units in constitutive SDS model with loss of p53 (E16.5 fetal liver; N = 5). CFU, colony forming unit; GEMM, granulocyte/erythroid/macrophage/ megakaryocyte; GM, granulocyte/macrophage; M, macrophage; G, granulocyte; BFU-E, burst forming unit-erythroid. *P*-values calculated using Wilcoxon Rank Sum Test comparing SDS models with (*Trp53* ^*+/–*^) and without p53 (*Trp53* ^*–/–*^). Horizontal lines indicate mean values. See S3 Table for complete statistics. **B,** Restored morphology and cell viability in the SDS mouse brain with loss of p53 (E14.5; VZ: ventricular zone). Scale bars for H&E sections represent 1000 μm (left panels) and 100 μm (right panels). Scale bars for TUNEL sections represent 50 μm. Yellow arrowheads highlight the zone of pronounced staining. **C,** Representative polysome traces illustrate that 80S peak levels remained low in mutant livers regardless of p53 status (E18.5, 100 μg RNA loaded).

In contrast to senescence and its molecular signature that included Tgfβ, p15^Ink4b^ and Myc, p53-dependence was not specific to the pancreas as loss of p53 impacted many phenotypes of the SDS mouse model. Although the lethality and growth impairment with reduced mass in the constitutive SDS mouse embryo was not improved (S1 Table; S1 Fig), loss of p53 had a restorative effect on blood progenitor levels (Fig 7A; S3 Table) and led to reduced apoptosis in the early SDS mouse brain to non-detectable levels (Fig 7B). As in the pancreas, polysome profiles are perturbed in *Sbds* ^*R126T/R126T*^ fetal livers, however loss of p53 resulted in only modest effects, with 80S monosome levels remaining far short of control levels (Figs 7C and S8).

## Discussion

Mutation of factors implicated in ribosome metabolism and translation lead to dramatic consequences for growth [3,8,9,67,68]. Our findings support the classification of SDS as a ribosomopathy [12,69]. Constitutive and targeted mouse models with SDS-associated Sbds alleles demonstrated severe growth impairment at both the organismal and organ levels. Mitogens were decreased in the SDS pancreas including low levels of the proto-oncogene Myc, a key regulator of exocrine pancreas expansion and acinar cell maintenance [70]. Moreover, arrest at the cell cycle level was evident with increased expression of cyclin inhibitors, senescence in the pancreas, and decreased BrdU-incorporation in the developing brain. With respect to direct evidence of a perturbation in ribosome metabolism, polysome analyses indicated a decrease in the proportion of 80S monosome levels in Sbds-ablated mutants relative to age-matched controls, with a notable difference in magnitude between the pancreas and liver. Although a subunit joining problem has been proposed previously in the context of Sbds mutations [12,21,37], our results are more consistent with an overall decrease in ribosome biogenesis, at least in the SDS pancreas. Finally, as observed in other ribosomopathies [54,65,71,72], we observed stabilization of p53 protein and increased Trp53 transcript levels in the SDS pancreas.

Constitutive ablation of Sbds in the mouse resulted in deficits in the hematopoietic and skeletal compartments, consistent with disease [25]. We previously demonstrated that targeted ablation of Sbds in the pancreas recapitulated all known SDS phenotypes of that organ [51]. Here we further identified a severe brain phenotype in constitutive models, with decreased proliferation in undifferentiated cells as well as pervasive cell death in differentiating neurons. These neural cell losses likely contribute to the perinatal lethality of the constitutive models. SDS is associated with cognitive impairment; imaging indicates reduced brain volume in patients [29,73] and approximately 20% of children with SDS meet criteria for intellectual disability [28] amongst other neurodevelopmental/behavioural concerns [27,28,74,75]. Neural cell death in the mouse occurred by p53-dependent apoptosis, consistent with neurological phenotypes observed in other ribosomopathy models [76–78].

Ribosome biogenesis and translational control have a significant impact on cell-cycle progression [79–81], therefore it is not surprising that cells with aberrant ribosome biogenesis and/or translation exhibit cell cycle arrest. However, while SDS-associated genotypes resulted in apoptosis in the fetal brain, senescence was observed in the postnatal pancreas. Given reports that Sbds loss may result in irregularities of the mitotic spindle [82,83], we considered that loss of Sbds could involve a DNA damage-induced senescence response. However, transcripts for DNA damage response factors Atm, Chek1 and Chek2 (S2 Table) were not upregulated. The senescence cell cycle arrest involved the p53/p21^Cip1^ and Tgfβ/p15^Ink4b^ networks. Tgfβ is a key member of the senescence-associated secretory phenotype, with roles in both the establishment and maintenance of senescence [57]. Select activation of the Smad effector proteins to relay the Tgfβ signalling cascade is context specific [84]; our findings indicated Smad2 is involved in mediating Tgfβ senescence in the SDS pancreas.

Senescence is considered a hallmark of premalignant tumours [85]. A Tgfβ/p15^Ink4b^-mediated senescent response has been observed in the context of tumour suppression in hepatocellular carcinoma human cell lines [45] and in lymphomas [44]. Senescence can, over time, promote malignant transformation in neighbouring cells due to the chronic secretion of inflammatory cytokines that are part of the senescence-associated secretory phenotype [86]. Although we have not observed tumour formation up to 14 months in our mice, acinar cells did express markers of dedifferentiation and in light of recent reports of early onset, aggressive pancreatic cancers in SDS patients [32,34], we argue that senescent cells present in the SDS pancreas could contribute to malignant transformation.

That senescence and the underlying pathway involving Tgfβ were not invoked in other organs of the constitutive SDS model highlighted the diversity of outcomes with loss of Sbds. At the same time, both pancreatic senescence and neural apoptosis were abrogated with genetic depletion of p53, implicating p53 as a key mediator of the response to Sbds loss in these two tissues. In the pancreas, we detected stabilization of p53 expression, detectable by 15 days of age, specifically in nuclei of acinar cells. Moreover, we discovered that the characteristic pancreatic phenotypes in SDS are extensively p53-dependent, including organ morphology and the shutdown of the zymogen granule proteome which was evident at the transcription level.

A recent zebrafish model of SDS, generated via morpholino-mediated knockdown of homolog *sbds* (*sbds*-MO), demonstrated deficits in pancreatic progenitor proliferation that were phenocopied by ablation of ribosomal constituent proteins, highlighting hypersensitivity of the pancreas compartment to mutations in ribosome-associated genes [40,87].

The absence of p53 did not constitute the rescue of overall growth or perinatal survival of the SDS mouse model highlighting p53-dependent and p53-independent aspects of SDS pathology (as were reported for the zebrafish model [40]). Specifically, the apparent improvements in the *Sbds/Trp53* pancreas double mutant phenotypes, were accompanied by a decrease in acinar cell size (a hallmark of translation insufficiency) supporting that protein synthesis remained compromised. Moreover, the profound 80S monosome loss in fetal liver cells (Fig 7) was not recovered with ablation of p53. We conclude that p53 is responding to Sbds deficiency by initiating cell cycle arrest (apoptosis or senescence) with some benefits. However, how the SDS-translation insufficiency triggers p53 activation, or how p53 activation achieves the apparent changes in phenotypes is not clear, perhaps through disturbed production or threshold shift of some critical checkpoint factor(s). With regard to the synthesis of specific proteins, we did note absence of recovery of amylase protein synthesis despite some resurgence of amylase transcript levels in the double mutant (Figs 5C and 6D).

In the pancreas, loss of *Sbds* is accompanied by cell cycle arrest and reduction of the zymogen granule transcriptome, leading to organ failure. Genetic ablation of *Trp53* attenuated the response with rescued growth and increased staining for dedifferentiation markers. It remains to be determined what alternative network(s) may signal the apoptosis that was subsequently observed in absence of p53. Overall, our findings indicate a cellular imperative to shut down cells with disrupted ribosome metabolism, consistent with reports of protective cell shutdown in other ribosomopathy models [6,71].

Can the study of the *Sbds*-deficient models inform a key question of what dictates organ hypersensitivity to ribosome dysfunction? Robust and ubiquitous expression argues against Sbds expression levels being the limiting factor directly underlying the varied organ responses in SDS [24,46]. The responses of organs to the SDS-translation deficiency varied in both timing and molecular signature. However, despite these differences, many aspects of the brain, blood and pancreas pathologies are all downstream of p53. Our study suggests that the perceived organ paucity in ribosomopathies stems in part from a disparity in molecular responses to translation dysfunction, likely downstream of p53 activation. Such responses, with dependence on cell type, can thus result in vastly different tissue outcomes.

## Materials and Methods

### Mice

All animal experiments were carried out under the guidelines of the Canadian Council on Animal Care, with approval of procedures by The Animal Care Committee of the Toronto Centre for Phenogenomics, Toronto, AUP #0093. The generation of constitutive SDS and SDS pancreas mouse models was described elsewhere [46,51]. Heterozygous carriers of either the missense (*R126T*) or null (–) mutation were indistinguishable from wildtype littermates. All mouse lines were maintained on a C57BL/J6 background and no gender effects were observed. Excision of the floxed *CKO* allele was achieved by breeding with the *Ptf1a* ^*Cre*^ mouse [88]. The p53 deficient strain B6.129S2-Trp53tm1Tyj/J (The Jackson Laboratory) was bred onto *Sbds* mutant lines for loss of p53 function studies. For embryonic staging, the morning of a vaginal plug was counted as embryonic day (E) 0.5. Mice were euthanized by decapitation, cervical dislocation or CO_2_ inhalation. Genotyping of DNA from tail samples was performed with the REDExtract-N-Amp Tissue PCR Kit (Sigma) using primers as previously described [51].

### Polysome analysis

Flash frozen tissues were lysed in Polysome Buffer (100 mM KCl, 5 mM MgCl_2_, 10 mM Tris–HCl pH9.0, 1% Triton X–100 and 1% sodium deoxycholate in diethylpyrocarbonate—treated water) on ice using a polytron. Insoluble cell debris was pelleted by centrifugation at 2,500 X g for 15 min at 4°C. Cyclohexamide (0.1 mg/mL) and heparin (1 mg/mL) were added to the supernatant, and equal amounts of RNA (determined by A_260_ using a Nanodrop Spectrophotometer) were loaded onto a 10–50% sucrose gradient (100 mM KCl, 5 mM MgCl_2_, 10 mM Tris-HCl pH9.0). Sucrose gradients were subjected to ultracentrifugation (151,000 X g for 2 hours at 4°C) prior to fractionation using a density gradient fractionation system (Brandel). UV absorbance (A_254_) was recorded using PeakTrak software (Teledyne Isco). Area under the curve (AUC) was calculated using Adobe Photoshop CS5.1 as described [89]. Individual peak/compartment areas were expressed relative to the total AUC of the profile.

### Histology and immunohistochemistry

For paraffin embedding, organs were dissected and fixed overnight in ice-cold 4% paraformaldehyde prior to processing into paraffin blocks. Sections with thickness of 5 μm were used. Safranin O (counterstained with Fast Green) staining was performed by the pathology core at the Toronto Centre for Phenogenomics. For immunohistochemistry, antigen retrieval was achieved by boiling in citrate buffer (10 mM sodium citrate, pH6.0), endogenous peroxidases were blocked with 6% H_2_O_2_, and non-specific epitopes were blocked with 5–10% goat serum. Antibodies used are given in S4 Table; antibody binding was visualized using diaminobenzidine reagent (Sigma). For senescence-associated β–galactosidase activity staining assays, fresh tissue was embedded and frozen in Tissue-Tek O.C.T. Compound (Sakura Finetek) as per supplier instructions. Frozen tissues were sectioned as 8 μm slices. Senescence staining was performed at pH5.5 as previously described [90]. Apoptosis was detected on paraffin sections by TUNEL assay either using the In Situ Cell Death Detection Kit (Roche) as per supplier’s instructions (fluorescein visualization) or by the pathology core at the Toronto Centre for Phenogenomics (diaminobenzidine visualization). 5–bromodeoxyuridine (50 μg/g, BD Biosciences) was injected in staged pregnant females 24 hours prior to embryo dissection at E14.5. 5–bromodeoxyuridine incorporation was detected using the BrdU In Situ Detection Kit (BD Biosciences). For cell size measurements, nuclei and acini from at least 3 non-overlapping micrographs taken at 40X magnification from 4 biological replicates were counted and measured.

### Electron microscopy

One week old pancreata were dissected and fixed in 2% glutaraldehyde in 0.1 M sodium cacodylate buffer (pH7.3). Fixed samples were processed and sectioned for electron microscopy by the joint Advanced Bioimaging Centre of The Hospital for Sick Children and Mount Sinai Hospital in Toronto.

### Analysis of fetal liver cells

Single cell suspensions from E14.5 embryo livers were prepared by grinding and filtering tissue through a 40 μm cell strainer (BD Biosciences). Cell suspensions were stained with conjugated antibodies against cell surface antigens with a FACSCalibur system (BD Biosciences) as previously described [91]. Antibodies used were Gr-1, c-kit (FITC-conjugated), Mac-1, and Ter119 (BD Biosciences). Flow cytometry data were analyzed using FlowJo software (Tree Star, Inc.).

### Colony forming assay for myeloid progenitor cells

Single cell suspensions from E16.5 embryo livers were prepared by grinding and filtering tissue through a 40 μm cell strainer (BD Biosciences). The number of cells per liver was determined by manual counting using a hemocytometer. Suspended cells (1X10^5^ in 0.3 ml Dulbecco’s Modified Eagle Media) were mixed with 3 ml of methylcellulose media (Stem Cell Technologies) containing recombinant murine stem cell factor, recombinant murine IL-3, recombinant human IL-6 and recombinant human erythropoietin (Stem Cell Technologies), split into thirds and plated on three 35 mm tissue culture plates. Cells were incubated for 7 days at 37°C, 5% CO_2_ and ≥95% humidity. Colonies of each cell type were identified and counted using a light microscope according to supplier’s instructions. Counts for all three plates of each cell type were averaged and presented as counts per 80,000 cells plated. At least five embryos of each genotype were investigated.

### Quantitative transcript analysis

Total RNA was isolated from RNAlater (QIAGEN) stabilized pancreas tissue (N = 3–4 for each genotype at each time point) or flash frozen tissues (brain, lung, liver, cartilage, bone; N = 4 for each genotype at each time point) using the RNeasy Mini Plus Kit (QIAGEN) according to manufacturer’s instructions with the addition of 5% β–mercaptoethanol in the homogenizing Buffer RLT Plus. For bone, cartilage and lung, homogenized tissues were first treated with Trizol (Life Technologies) before application to the RNeasy spin columns. Quality control and real-time quantitative PCR was performed as previously described [51]. Results are presented relative to the expression of the optimal control gene (four genes tested for each sample) for that tissue and time point as determined by GeNORM analysis [92]. A significant change was defined as a ≥2 fold difference with a *P*–value <0.05. Oligonucleotide primers are given in S5 Table. Expression levels of 84 cellular–senescence associated genes were assayed using the SABiosciences Cellular Senescence RT^2^ Profiler PCR Array (QIAGEN) with total RNA isolated from pancreata of mice at 15 and 25 days of age. A significant change was defined, as per supplier’s instructions, as a ≥3 fold difference with a *P*-value of <0.05. Selected gene results were confirmed by real-time quantitative PCR of independently prepared cDNA samples with distinct primer sets (with the exception of Cdkn2b where QIAGEN array primers were used).

### Immunoblotting

Pancreas tissue (~30 mg) from 20 day old mice (prior to fat infiltration) was homogenized in RIPA buffer (150 mM NaCl, 1% NP-40, 0.5% sodium deoxycholate, 0.1% sodium dodecyl sulfate, 50 mM Tris-HCl, pH7.5) using a polytron over ice. Insoluble components were pelleted by centrifugation (17,000 X g at 4°C). Equal amounts of protein (determined by Lowry assay, BioRad) in 2X Laemmli buffer were separated by 12% SDS–PAGE and either stained with silver salts or Coomassie brilliant blue, or blotted using the Trans–Blot Turbo Transfer Pack with the Trans-Blot Turbo Transfer System (BioRad). Trans-Blot Turbo nitrocellulose membranes (BioRad) were blocked in 5% (w/v) powdered skim milk (5% (w/v) goat serum for Novacastra CM5 p53 antibody) prior to overnight incubation with primary antibodies followed by species appropriate horseradish peroxidase-conjugated secondary antibodies (S4 Table). Bound antibodies were visualized with Amersham ECL Prime Western Blotting Detection Reagent (GE Healthcare Life Sciences) on the ChemiDoc MP Imaging System using Image-Lab 4.1 Software (BioRad).

### Statistical methods

All statistical tests were carried out using R statistical software (R Foundation, from http://www.r-project.org). For T-tests, Welch’s correction was used to adjust for non-constant variance. Wilcoxon Rank Sum Test and Kruskal-Wallis analysis of variance were used where data did not show normal distribution. Bonferroni adjusted critical values were used to declare significance, adjusting for the number of comparisons per analysis. Raw *P*-values are reported.

## Supporting Information

S1 Fig SDS embryos display impaired growth. **A,** Loss of Sbds function resulted in decreased mass. In comparisons, *controls* refers to the grouping of *Sbds* ^*+/+*^ and *Sbds* ^*R126T/+*^ genotypes. Loss of p53 did not impact mutant mass (**B**) but did restore mutant embryo length (**C**). Embryos were weighed and measured at E18.5 and are shown as mean±SD. Length was calculated relative to a chosen control mouse whose length was set as 1. Pairwise differences were evaluated using the Wilcoxon Rank Sum Test.
(TIF)

S2 Fig SDS embryo lungs express lung differentiation markers but display limited alveolar space. **A,** Histochemistry at E18.5 of *Sbds* ^*R126T/+*^ and *Sbds* ^*R126T/R126T*^ lung tissue revealed an absence of alveolar spaces. Immunohistochemistry demonstrated expression of columnar epithelial cell differentiation marker clara cell 10 (CC10) and pulmonary alveoli type-2 cell marker prosurfactant protein C (proSP-c) in mutant tissue. Loss of p53 did not have a significant impact. **B,** Quantitative transcript analysis of lung total RNA at E18.5. Fold change: *Sbds* ^*R126T/R126T*^/*Sbds* ^*R126T/+*^; N = 4. Criteria for significance: ≥2 fold change, *P*<0.05. Expression is relative to Gusb.
(TIF)

S3 Fig Myeloid progenitor levels by colony forming assay are low in SDS embryos. Colony forming assays of fetal (E16.5) liver cells showed decreased growth of all blood lineage progenitors in both SDS mouse models indicating impaired hematopoiesis, N = 5 for each genotype. Error bars represent SD. CFU, colony forming unit; GEMM, granulocyte/erythroid/macrophage/ megakaryocyte; GM, granulocyte/macrophage; M, macrophage; G, granulocyte; BFU-E, burst forming unit-erythroid.
(TIF)

S4 Fig Myeloid progenitor levels by cytometry are low in SDS embryos. **A,** Cytometry analysis of Mac-1^+^/Gr-1^+^ and Gr-1^+^/c-Kit^-^ cells indicated decreased numbers of granulocyte precursors in the compound heterozygote model (*Sbds* ^*R126T/-*^) in fetal livers at E14.5. Error bars represent ±SEM, *P*-values calculated using T-test. **B,** Cytometry analysis of Ter119^+^ cells at E18.5 showed no change in erythrocyte precursors in either mutant model. Error bars represent ±SEM, *P*-values calculated using T-test.
(TIF)

S5 Fig Neuronal pathology progresses to pervasive necrosis by E18.5 in SDS models. H&E staining of sagittal brain sections of E18.5 mutant embryo showed reduced tissue mass with severe necrosis, notably evident in the pallium region shown in the expanded lower panels. A littermate control is shown for comparison. Scale bar represents 1000 μm.
(TIF)

S6 Fig Bone marrow and liver do not show overt apoptosis with loss of Sbds at E18.5 Low numbers of apoptotic nuclei (dark brown; examples indicated with yellow arrowheads), identified by TUNEL assay were evident in liver and marrow tissues of *Sbds* ^*R126T/R126T*^ (**A**) and *Sbds* ^*R126T/-*^ (**B**) models and their respective littermate controls. Scale bar represents 100 μm.
(TIF)

S7 Fig Genetic ablation of Trp53 abrogated senescence-associated β-galatosidase activity in the SDS pancreas. The β-galatosidase activity detected in acini of the SDS pancreas (see Fig 3) was abrogated with genetic ablation of *Trp53*. Littermates are shown at 32 days of age, scale bars represent 100 μm.
(TIF)

S8 Fig Pancreas and liver demonstrate reduced 80S monosome levels. The area under the curve (AUC) was calculated and averaged for ribosome subunits, monosomes and polysomes for the pancreas (20 days of age) and fetal liver (E18.5). *P*-values were calculated assuming polysome profiles are identically distributed within a genotype category using Welch’s T-test.
(TIF)

S1 Table Lethality with *Sbds* ^*R126T*^ alleles. Breeding of mice that were heterozygous for SDS-associated alleles did not yield live mice that were homozygous for SDS-associated alleles, although adherence to Mendelian ratios was evident prior to full gestation (E18.5). Ablation of p53 did not resolve the lethality of the SDS model mice at birth.
(DOCX)

S2 Table Cellular Senescence PCR Array. Expression levels of 84 cellular-senescence associated genes were assayed using the SABiosciences Cellular Senescence RT^2^ Profiler PCR Array (QIAGEN) with total pancreata RNA of mice at 15 and 25 days of age. Fold change indicated corresponds to *Sbds* ^*P-/R126T*^ / *Sbds* ^*P-/+*^. A significant change was defined, as per supplier’s instructions, as ≥3 fold difference, *P*-value of <0.05 (Student’s *T*-test). Red bold: down-regulation; blue bold: up-regulation. Gene groupings are as designated by array supplier. Raw Ct values are available upon request.
(DOCX)

S3 Table Myeloid progenitors of SDS embryos levels improve in the absence of p53. Myeloid progenitors were counted following cultivation of equal cell numbers of single organ suspensions of fetal livers of mutant and control embryos at E16.5. N corresponds to the number of embryos analysed for each indicated genotype.
(DOCX)

S4 Table Antibodies used in this study. Sources of all primary and secondary antibodies used in immunohistochemistry and immunoblotting procedures are listed.
(DOCX)

S5 Table Oligonucleotides used in this study. Sequences of all oligonucleotide primers used for the gene expression studies are listed.
(DOCX)
