# Supplementary figures and images for: In Vivo Senescence in the Sbds-Deficient Murine Pancreas: Cell-Type Specific Consequences of Translation Insufficiency
Source: PLoS Genet. 2015 Jun 9;11(6):e1005288. doi: 10.1371/journal.pgen.1005288 (PMC4461263; doi:10.1371/journal.pgen.1005288)

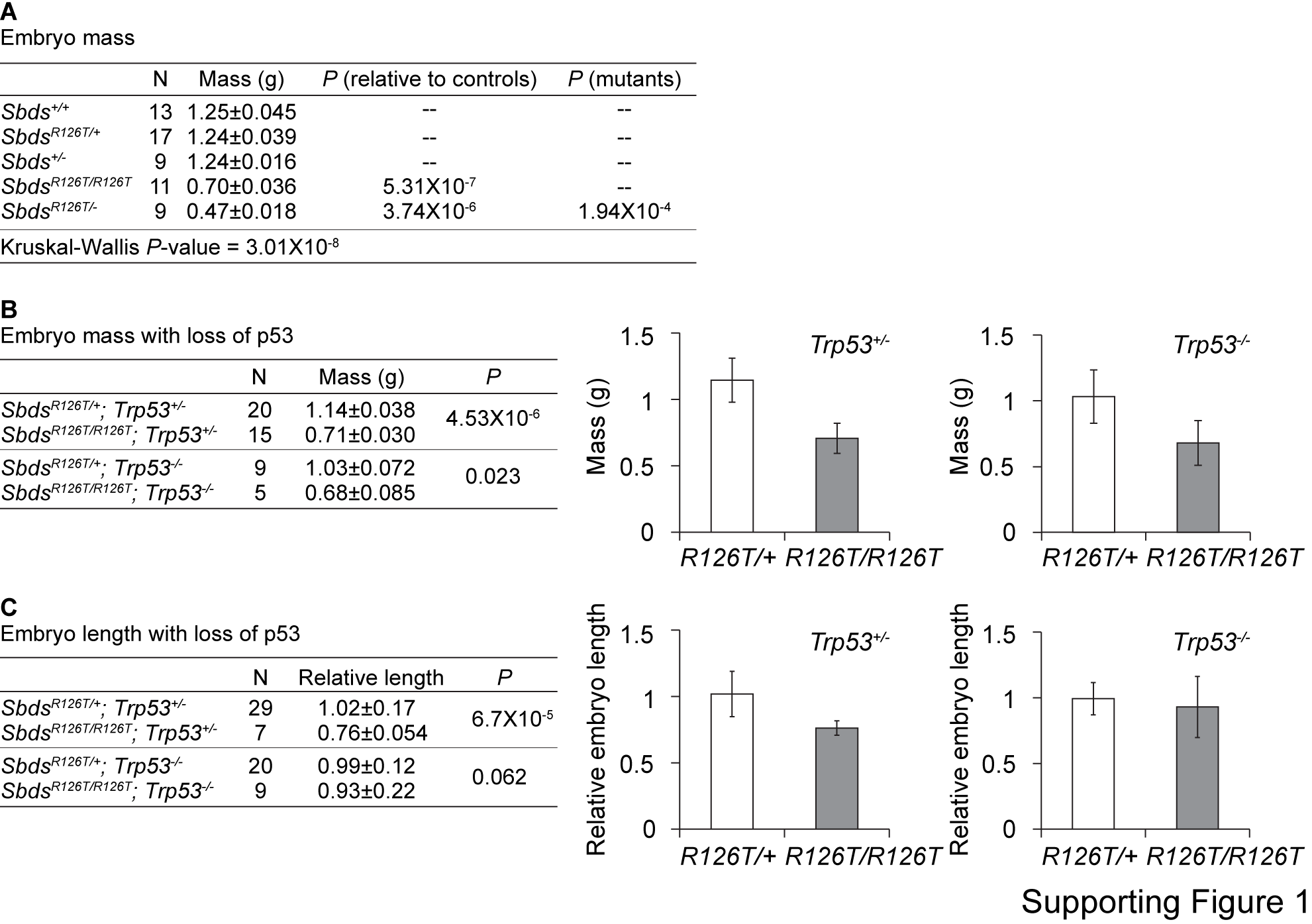

Supplement: S1 Fig — A, Loss of Sbds function resulted in decreased mass. In comparisons, controls refers to the grouping of Sbds +/+ and Sbds R126T/+ genotypes. Loss of p53 did not impact mutant mass (B) but did restore mutant embryo length (C). Embryos were weighed and measured at E18.5 and are shown as mean±SD. Length was calculated relative to a chosen control mouse whose length was set as 1. Pairwise differences were evaluated using the Wilcoxon Rank Sum Test. (TIF) [file pgen.1005288.s001.tif]

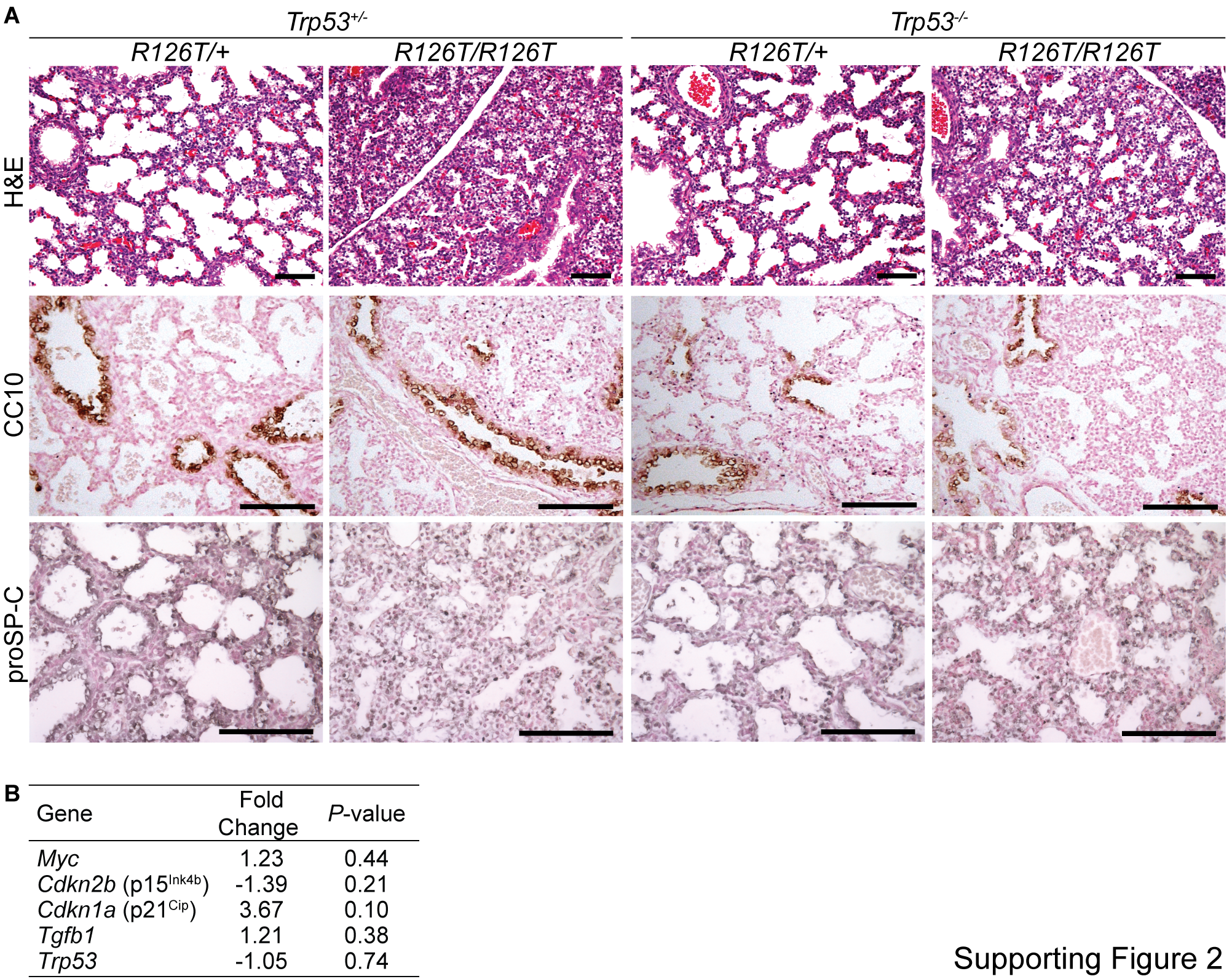

Supplement: S2 Fig — A, Histochemistry at E18.5 of Sbds R126T/+ and Sbds R126T/R126T lung tissue revealed an absence of alveolar spaces. Immunohistochemistry demonstrated expression of columnar epithelial cell differentiation marker clara cell 10 (CC10) and pulmonary alveoli type-2 cell marker prosurfactant protein C (proSP-c) in mutant tissue. Loss of p53 did not have a significant impact. B, Quantitative transcript analysis of lung total RNA at E18.5. Fold change: Sbds R126T/R126T/Sbds R126T/+; N = 4. Criteria for significance: ≥2 fold change, P<0.05. Expression is relative to Gusb. (TIF) [file pgen.1005288.s002.tif]

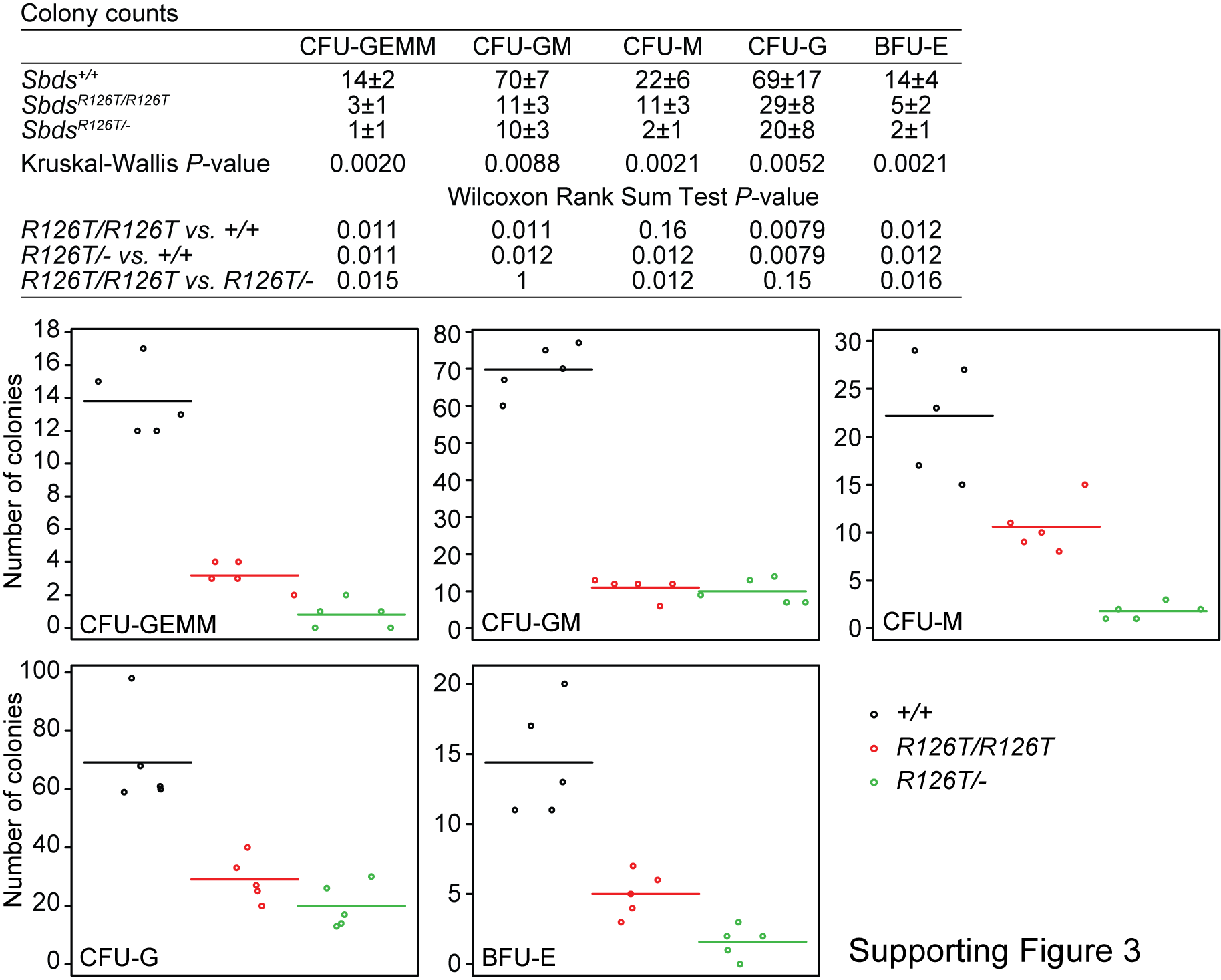

Supplement: S3 Fig — Colony forming assays of fetal (E16.5) liver cells showed decreased growth of all blood lineage progenitors in both SDS mouse models indicating impaired hematopoiesis, N = 5 for each genotype. Error bars represent SD. CFU, colony forming unit; GEMM, granulocyte/erythroid/macrophage/ megakaryocyte; GM, granulocyte/macrophage; M, macrophage; G, granulocyte; BFU-E, burst forming unit-erythroid. (TIF) [file pgen.1005288.s003.tif]

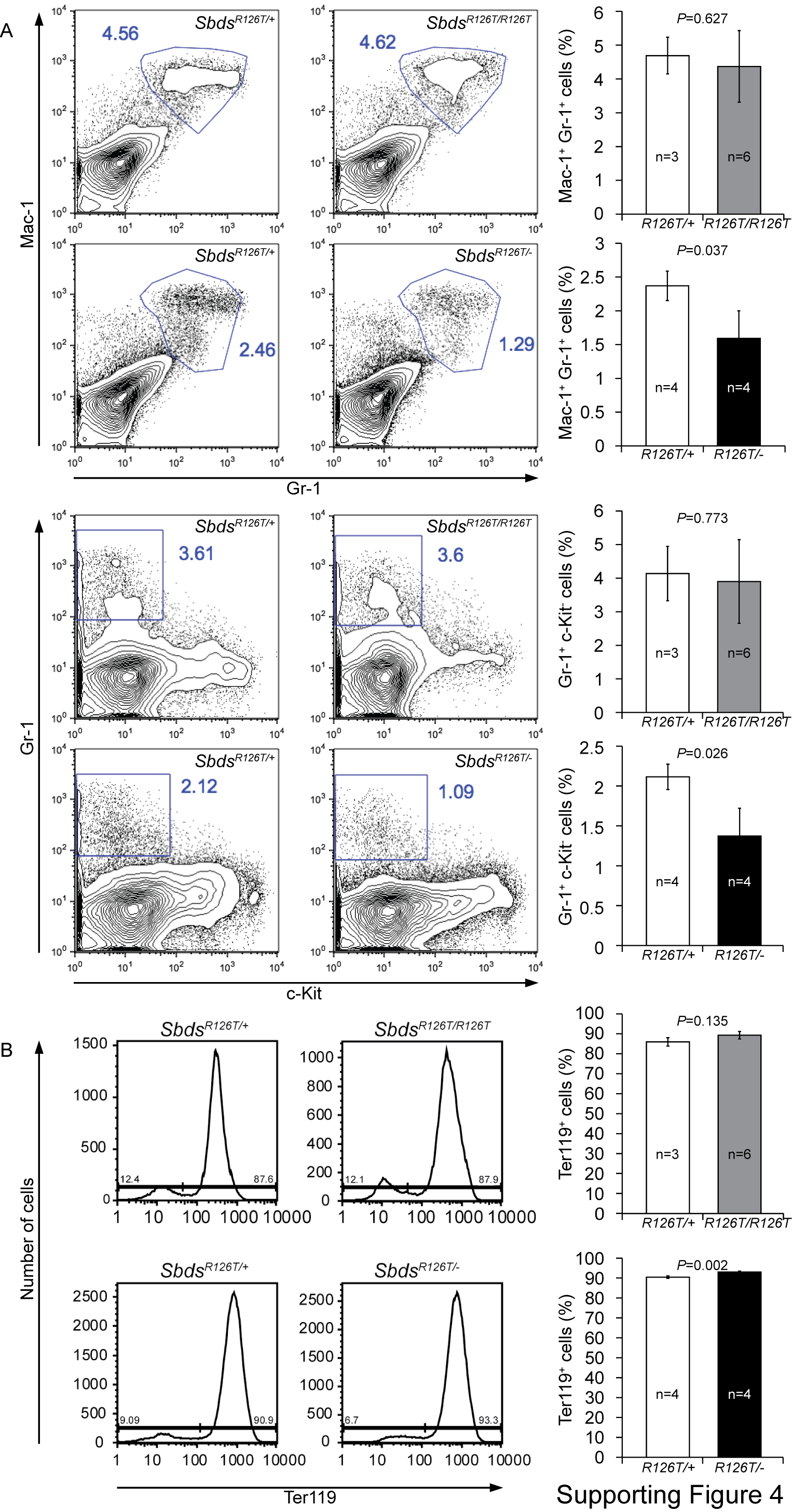

Supplement: S4 Fig — A, Cytometry analysis of Mac-1+/Gr-1+ and Gr-1+/c-Kit- cells indicated decreased numbers of granulocyte precursors in the compound heterozygote model (Sbds R126T/-) in fetal livers at E14.5. Error bars represent ±SEM, P-values calculated using T-test. B, Cytometry analysis of Ter119+ cells at E18.5 showed no change in erythrocyte precursors in either mutant model. Error bars represent ±SEM, P-values calculated using T-test. (TIF) [file pgen.1005288.s004.tif]

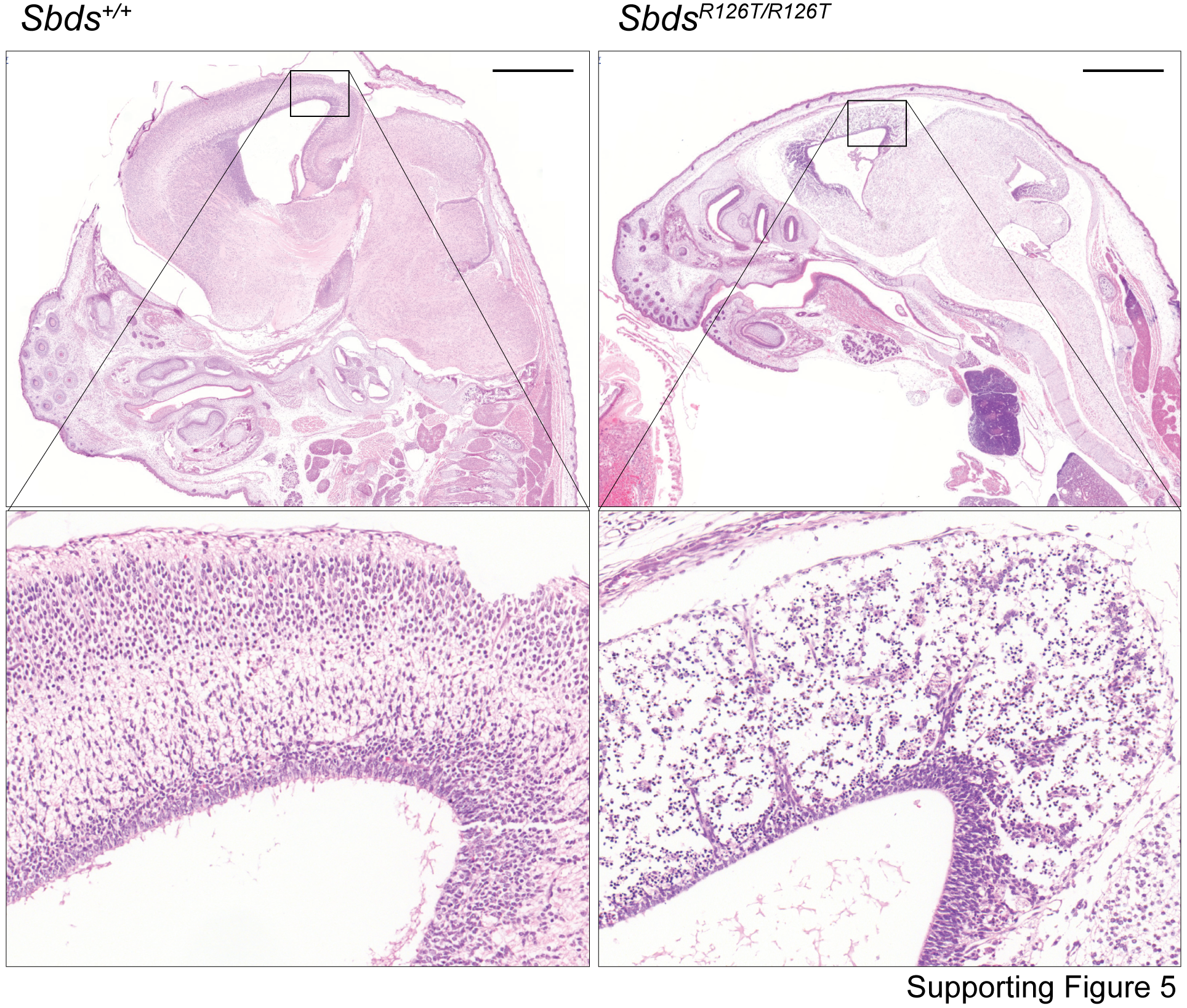

Supplement: S5 Fig — H&E staining of sagittal brain sections of E18.5 mutant embryo showed reduced tissue mass with severe necrosis, notably evident in the pallium region shown in the expanded lower panels. A littermate control is shown for comparison. Scale bar represents 1000 μm. (TIF) [file pgen.1005288.s005.tif]

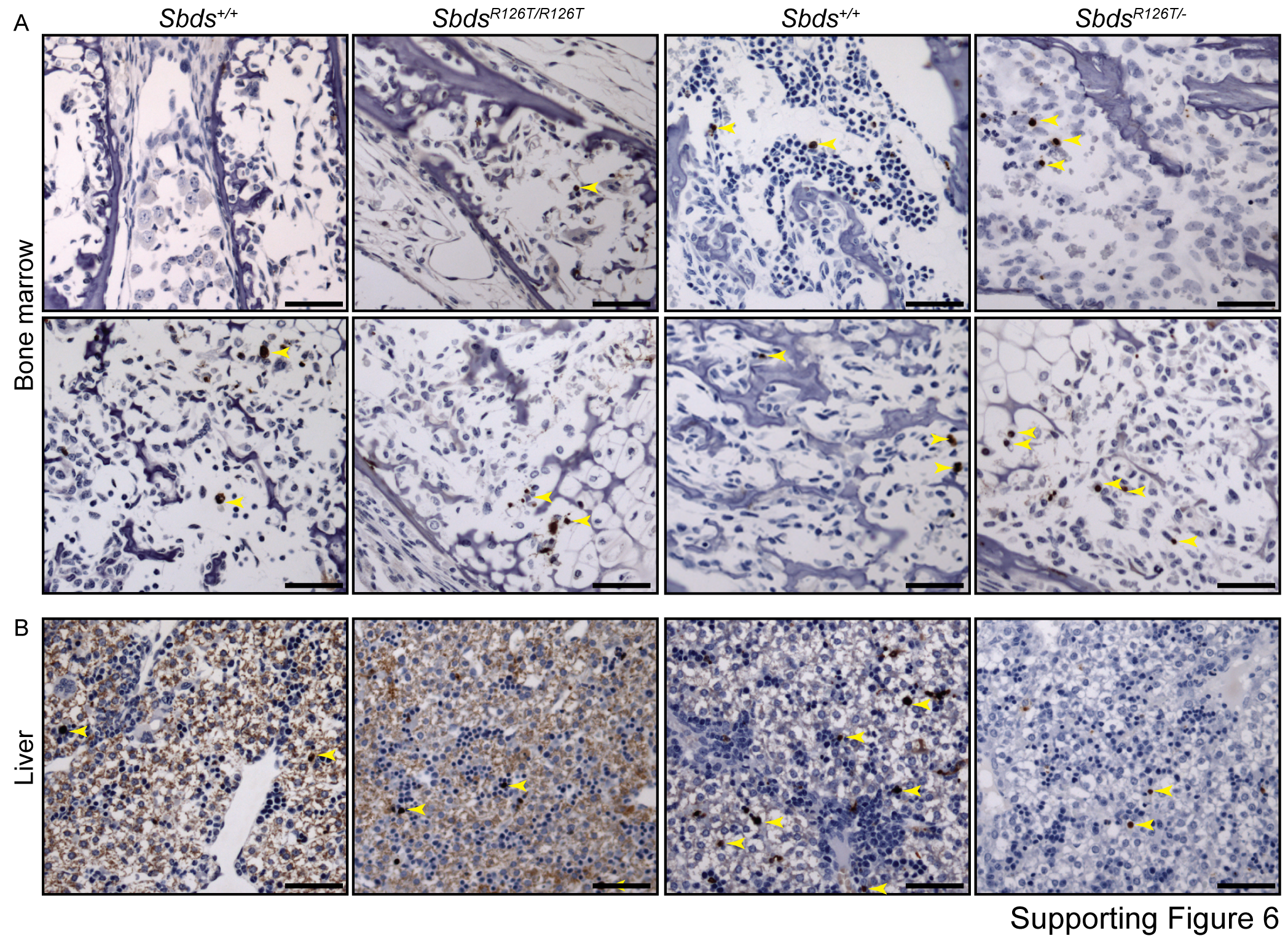

Supplement: S6 Fig — Low numbers of apoptotic nuclei (dark brown; examples indicated with yellow arrowheads), identified by TUNEL assay were evident in liver and marrow tissues of Sbds R126T/R126T (A) and Sbds R126T/- (B) models and their respective littermate controls. Scale bar represents 100 μm. (TIF) [file pgen.1005288.s006.tif]

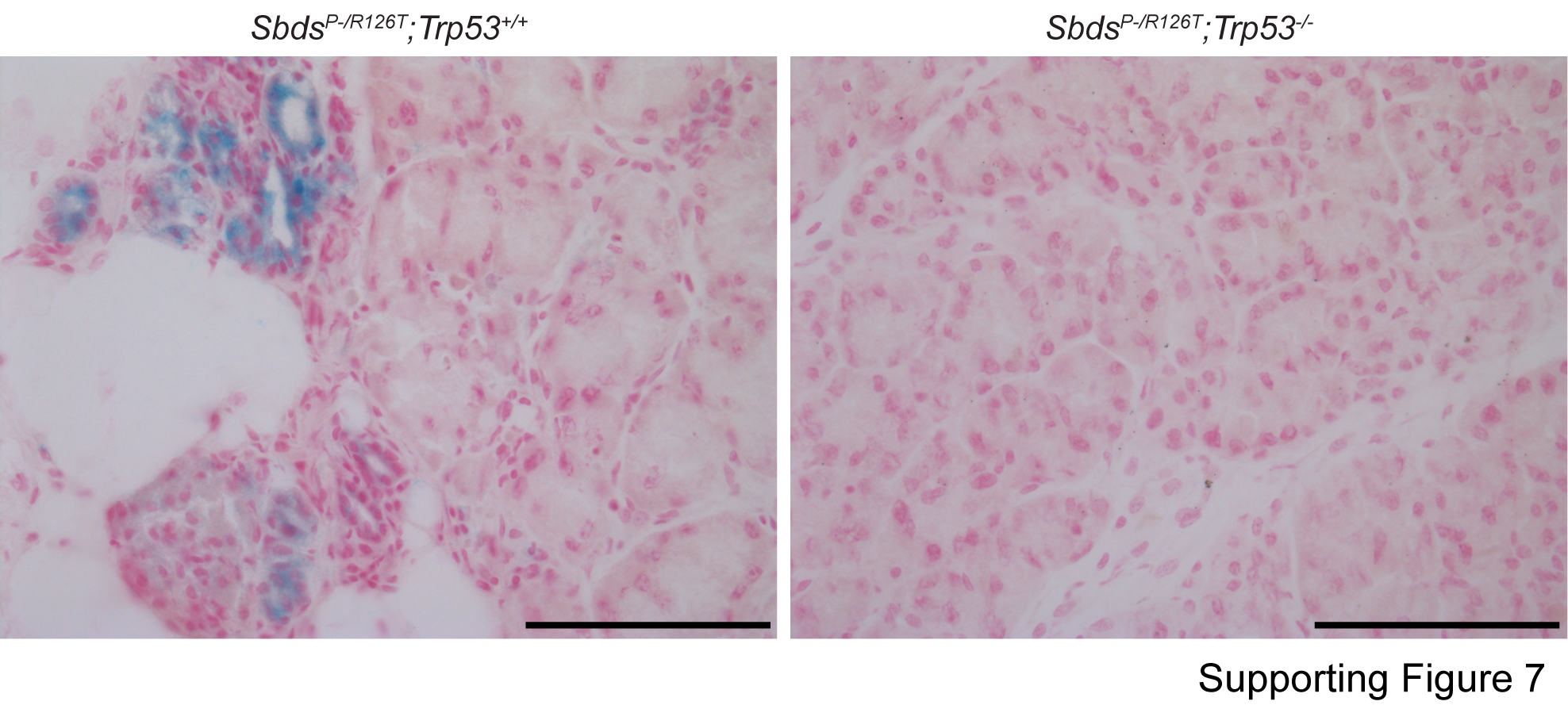

Supplement: S7 Fig — The β-galatosidase activity detected in acini of the SDS pancreas (see Fig 3) was abrogated with genetic ablation of Trp53. Littermates are shown at 32 days of age, scale bars represent 100 μm. (TIF) [file pgen.1005288.s007.tif]

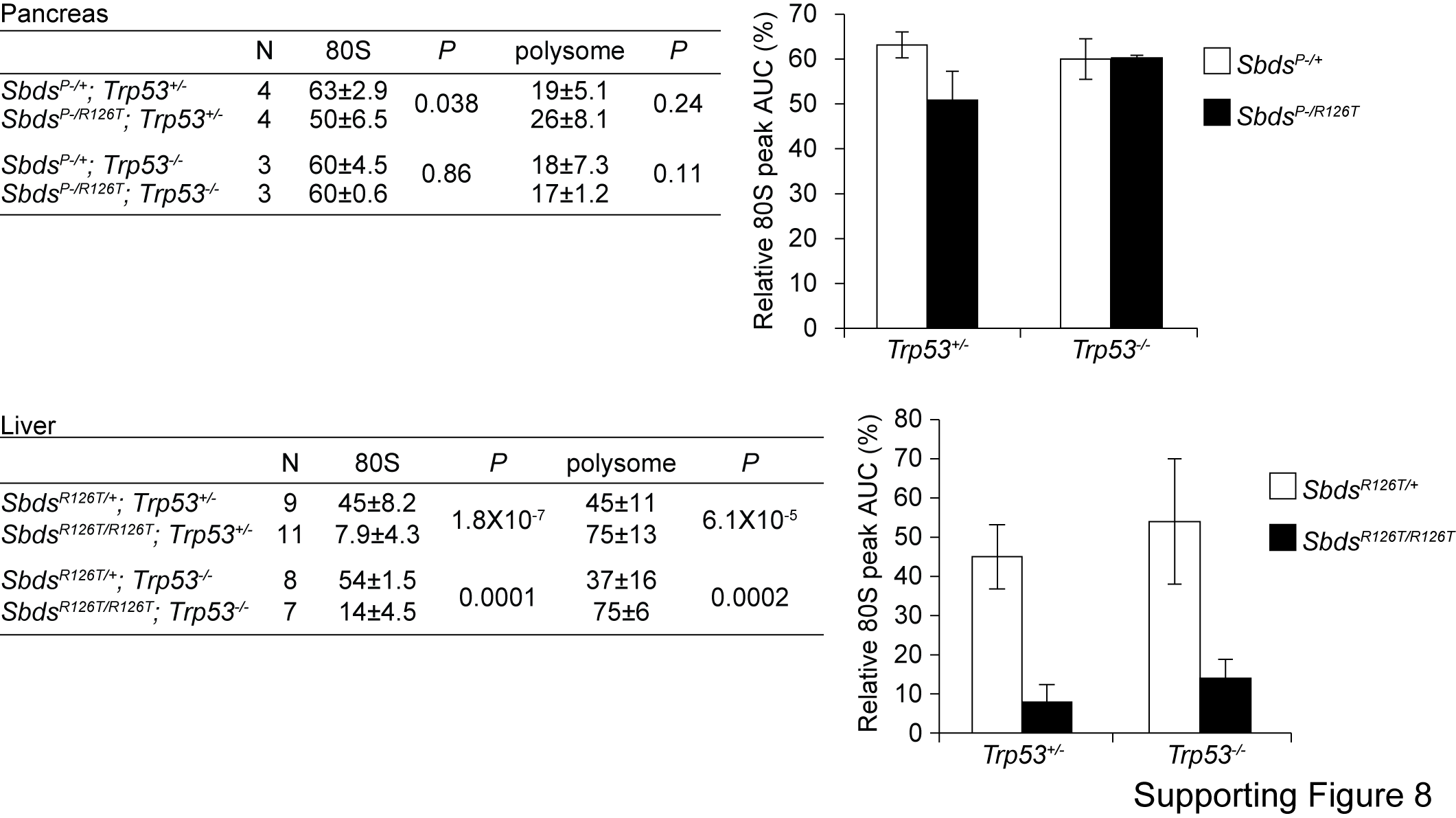

Supplement: S8 Fig — The area under the curve (AUC) was calculated and averaged for ribosome subunits, monosomes and polysomes for the pancreas (20 days of age) and fetal liver (E18.5). P-values were calculated assuming polysome profiles are identically distributed within a genotype category using Welch’s T-test. (TIF) [file pgen.1005288.s008.tif]
