# Supplementary material for: In Vivo Senescence in the Sbds-Deficient Murine Pancreas: Cell-Type Specific Consequences of Translation Insufficiency
Source: PLoS Genet. 2015 Jun 9;11(6):e1005288. doi: 10.1371/journal.pgen.1005288 (PMC4461263; doi:10.1371/journal.pgen.1005288)
Supplement: S1 Table — Breeding of mice that were heterozygous for SDS-associated alleles did not yield live mice that were homozygous for SDS-associated alleles, although adherence to Mendelian ratios was evident prior to full gestation (E18.5). Ablation of p53 did not resolve the lethality of the SDS model mice at birth. (DOCX) [file pgen.1005288.s009.docx]

**Supporting Table 1. Lethality with *Sbds^R126T^* alleles**

|  | Expected | |  | | Observed | | | |
| --- | --- | --- | --- | --- | --- | --- | --- | --- |
|  | Odds | Percent |  | Embryos (E18.5) | | Percent | Live Births | Percent |
| *Sbds^+/+^; Trp53^+/+^* | 1/16 | 6% |  | 5 | | 3% | 7 | 6% |
| *Sbds^+/+^; Trp53^+/-^* | 1/8 | 13% |  | 35 | | 18% | 24 | 20% |
| *Sbds^+/+^; Trp53^-/-^* | 1/16 | 6% |  | 7 | | 4% | 4 | 3% |
| *Sbds^R126T/+^; Trp53^+/+^* | 1/8 | 13% |  | 8 | | 4% | 13 | 11% |
| *Sbds^R126T/+^; Trp53^+/-^* | 1/4 | 25% |  | 71 | | 36% | 62 | 51% |
| *Sbds^R126T/+^; Trp53^-/-^* | 1/8 | 13% |  | 17 | | 9% | 11 | 9% |
| *Sbds^R126T/R126T^; Trp53^+/+^* | 1/16 | 6% |  | 5 | | 3% | 0 | 0% |
| *Sbds^R126T/R126T^; Trp53^+/-^* | 1/8 | 13% |  | 32 | | 16% | 0 | 0% |
| *Sbds^R126T/R126T^; Trp53^-/-^* | 1/16 | 6% |  | 15 | | 8% | 0 | 0% |
|  |  | TOTAL | | 195 | | TOTAL | 121 |  |

We previously showed that embryos homozygous for a null allele of Sbds arrest at E6.5^†^.

^†^Zhang, S. *et al.* Loss of the mouse ortholog of the shwachman-diamond syndrome gene (*Sbds*) results in early embryonic lethality. *Molecular and cellular biology* 26, 6656-6663 (2006).
