# Supplementary material for: In Vivo Senescence in the Sbds-Deficient Murine Pancreas: Cell-Type Specific Consequences of Translation Insufficiency
Source: PLoS Genet. 2015 Jun 9;11(6):e1005288. doi: 10.1371/journal.pgen.1005288 (PMC4461263; doi:10.1371/journal.pgen.1005288)
Supplement: S2 Table — Expression levels of 84 cellular-senescence associated genes were assayed using the SABiosciences Cellular Senescence RT2 Profiler PCR Array (QIAGEN) with total pancreata RNA of mice at 15 and 25 days of age. Fold change indicated corresponds to Sbds P-/R126T / Sbds P-/+. A significant change was defined, as per supplier’s instructions, as ≥3 fold difference, P-value of <0.05 (Student’s T-test). Red bold: down-regulation; blue bold: up-regulation. Gene groupings are as designated by array supplier. Raw Ct values are available upon request. (DOCX) [file pgen.1005288.s010.docx]

**Supporting Table 2. Cellular senescence PCR array**

|  |  |  |  |  | **Gene groupings by function** | | | | | | | | | | | | |
| --- | --- | --- | --- | --- | --- | --- | --- | --- | --- | --- | --- | --- | --- | --- | --- | --- | --- |
|  |  |  |  |  | **Senescence pathway** | **Initiators** | | | | | | **Responses** | | | | | |
|  |  |  |  |  |  | **p53/pRb signalling** | **Interferon related** | **Insulin growth factor related** | **MAPK signalling** | **Oxidative stress** | **DNA damage** | **p53 effectors** | **p21 Effectors** | **p16 effectors** | **Cytoskeleton related** | **Cell Adhesion** | **Other (senescence responses)** |
|  |  |  |  |  |  |  |  |  |  |  |  |  |  |  |  |  |  |
|  |  |  |  |  |  |  |  |  |  |  |  |  |  |  |  |  |  |
|  | **15 days of age** | | **25 days of age** | |  |  |  |  |  |  |  |  |  |  |  |  |  |
| **Gene** | **Fold Change** | ***P*** | **Fold Change** | ***P*** |  |  |  |  |  |  |  |  |  |  |  |  |  |
| Abl1 | -1.04 | 0.848 | -1.59 | 0.095 |  | **•** |  |  |  |  |  |  |  |  |  |  |  |
| Akt1 | -1.96 | 0.119 | -3.69 | 0.019 |  | **•** |  |  |  |  |  |  |  |  | **•** |  |  |
| Aldh1a3 | 2.73 | 0.062 | -1.61 | 0.069 |  | **•** | **•** |  |  | **•** |  | **•** |  |  |  |  |  |
| Atm | -1.66 | 0.031 | -2.51 | 0.033 | **•** |  |  |  |  |  | **•** |  |  |  |  |  |  |
| Bmi1 | -1.01 | 0.986 | -2.31 | 0.159 | **•** |  |  |  |  |  |  |  |  |  |  |  |  |
| Calr | -1.82 | 0.015 | -3.03 | 0.062 |  |  |  |  |  |  |  |  | **•** |  |  |  |  |
| Ccna2 | -2.35 | 0.058 | -2.36 | 0.178 |  | **•** |  |  |  |  |  |  |  |  |  |  |  |
| Ccnb1 | -2.19 | 0.012 | -1.80 | 0.229 |  | **•** |  |  |  |  |  |  |  |  |  |  |  |
| Ccnd1 | 1.34 | 0.200 | 2.21 | 0.019 | **•** |  |  |  |  |  |  |  |  |  |  |  |  |
| Ccne1 | -1.80 | 0.190 | -1.49 | 0.152 | **•** |  |  |  |  |  |  |  |  |  |  |  |  |
| Cd44 | 1.27 | 0.225 | 3.59 | 0.003 |  |  |  |  |  |  |  |  |  |  |  | **•** |  |
| Cdc25c | -2.38 | 0.049 | -3.54 | 0.163 |  | **•** |  |  |  |  |  |  |  |  |  |  |  |
| Cdk2 | -1.10 | 0.658 | 1.31 | 0.284 | **•** |  |  |  |  |  |  |  |  |  |  |  |  |
| Cdk4 | -1.76 | 0.087 | -1.68 | 0.001 | **•** |  |  |  |  |  |  |  |  |  |  |  |  |
| Cdk6 | -1.41 | 0.389 | -2.04 | 0.133 | **•** |  |  |  |  |  |  |  |  |  |  |  |  |
| Cdkn1a | 3.77 | 0.042 | 1.62 | 0.485 | **•** |  | **•** |  |  |  |  |  |  |  |  |  |  |
| Cdkn1b | -1.35 | 0.244 | -2.20 | 0.003 |  |  | **•** |  |  |  |  |  |  |  |  |  |  |
| Cdkn1c | 1.46 | 0.247 | 1.51 | 0.072 |  | **•** |  |  |  |  |  |  |  |  |  |  |  |
| Cdkn2a | 2.58 | 0.020 | -1.13 | 0.662 | **•** |  |  |  |  |  |  |  |  |  |  |  |  |
| Cdkn2b | 4.06 | 0.057 | 4.90 | 0.017 |  | **•** |  |  |  |  |  |  |  |  |  |  |  |
| Cdkn2c | 1.00 | 0.983 | 1.30 | 0.374 |  | **•** |  |  |  |  |  |  |  |  |  |  |  |
| Cdkn2d | 1.02 | 0.905 | 1.46 | 0.446 | **•** |  |  |  |  |  |  |  |  |  |  |  |  |
| Chek1 | -2.03 | 0.073 | -1.33 | 0.065 | **•** |  |  |  |  |  |  |  |  |  |  |  |  |
| Chek2 | -1.19 | 0.820 | -2.95 | 0.020 | **•** |  |  |  |  |  |  |  |  |  |  |  |  |
| Cited2 | -1.21 | 0.568 | -2.35 | 0.072 |  | **•** |  |  |  |  |  |  |  |  |  |  |  |
| Col1a1 | 2.58 | 0.068 | 4.43 | 0.019 |  |  |  |  |  |  |  |  |  |  |  | **•** |  |
| Col3a1 | 1.83 | 0.066 | 2.36 | 0.054 |  |  |  |  |  |  |  |  |  |  |  | **•** |  |
| Creg1 | -2.25 | 0.043 | -3.08 | 0.001 |  | **•** |  |  |  |  |  |  |  |  |  |  |  |
| E2f1 | -1.67 | 0.265 | -1.34 | 0.334 | **•** |  |  |  |  |  |  | **•** |  |  |  |  |  |
| E2f3 | 1.26 | 0.371 | 1.74 | 0.071 | **•** |  |  |  |  |  |  |  |  |  |  |  |  |
| Egr1 | 3.39 | 0.019 | 4.31 | 0.037 |  |  | **•** |  |  |  |  |  |  |  |  |  |  |
| Ets1 | 1.43 | 0.642 | 15.52 | 0.006 | **•** |  |  |  |  |  |  |  |  |  |  |  |  |

| (continued) | |  |  |  | **Gene groupings by function** | | | | | | | | | | | | |
| --- | --- | --- | --- | --- | --- | --- | --- | --- | --- | --- | --- | --- | --- | --- | --- | --- | --- |
|  |  |  |  |  | **Senescence pathway** | **Initiators** | | | | | | **Responses** | | | | | |
|  |  |  |  |  |  | **p53/pRb signalling** | **Interferon related** | **Insulin growth factor related** | **MAPK signalling** | **Oxidative stress** | **DNA damage** | **p53 effectors** | **p21 Effectors** | **p16 effectors** | **Cytoskeleton related** | **Cell Adhesion** | **Other (senescence responses)** |
|  |  |  |  |  |  |  |  |  |  |  |  |  |  |  |  |  |  |
|  |  |  |  |  |  |  |  |  |  |  |  |  |  |  |  |  |  |
|  | **15 days of age** | | **25 days of age** | |  |  |  |  |  |  |  |  |  |  |  |  |  |
| **Gene** | **Fold Change** | ***P*** | **Fold Change** | ***P*** |  |  |  |  |  |  |  |  |  |  |  |  |  |
| Ets2 | -1.79 | 0.126 | -2.01 | 0.129 | **•** |  |  |  |  |  |  |  |  |  |  |  |  |
| Fn1 | 3.42 | 0.012 | 3.78 | 0.002 |  |  |  |  |  |  |  |  |  |  | **•** |  |  |
| Gadd45a | 1.71 | 0.025 | 1.65 | 0.263 |  |  |  |  |  |  | **•** |  |  |  |  |  |  |
| Glb1 | -1.39 | 0.210 | -1.31 | 0.478 |  |  |  |  |  |  |  |  |  |  |  |  | **•** |
| Gsk3b | 1.13 | 0.402 | -1.40 | 0.233 |  | **•** |  |  |  |  |  |  |  |  |  |  |  |
| Hras1 | -2.00 | 0.030 | -3.96 | 0.005 |  |  |  |  | **•** | **•** |  | **•** |  |  | **•** |  |  |
| Id1 | 1.11 | 0.524 | 2.06 | 0.032 |  | **•** |  |  |  |  |  |  |  |  |  |  |  |
| Ifng | -2.75 | 0.161 | -8.44 | 0.007 |  |  | **•** |  |  |  |  |  |  |  |  |  |  |
| Igf1 | -1.08 | 0.843 | -1.42 | 0.176 |  |  |  | **•** |  |  |  |  |  |  |  |  |  |
| Igf1r | -1.48 | 0.042 | -1.49 | 0.451 |  | **•** |  | **•** |  |  |  |  |  |  |  |  |  |
| Igfbp3 | 1.31 | 0.369 | 1.19 | 0.830 |  | **•** | **•** | **•** |  |  |  | **•** |  |  |  |  |  |
| Igfbp5 | 2.04 | 0.127 | 4.66 | 0.010 |  |  |  | **•** |  |  |  |  |  |  |  |  |  |
| Igfbp7 | 1.89 | 0.031 | 3.20 | 1X10^-4^ |  |  | **•** | **•** |  |  |  |  |  |  |  |  |  |
| Ing1 | -1.25 | 0.389 | -2.63 | 0.079 |  | **•** |  |  |  |  |  |  |  |  |  |  |  |
| Irf3 | -1.57 | 0.418 | -4.85 | 0.008 |  |  | **•** |  |  |  |  |  |  |  |  |  |  |
| Irf5 | 3.80 | 0.015 | 5.31 | 0.001 |  |  | **•** |  |  |  |  |  |  |  |  |  |  |
| Irf7 | 3.36 | 0.018 | 3.07 | 0.027 |  |  | **•** |  |  |  |  |  |  |  |  |  |  |
| Map2k1 | -1.06 | 0.710 | 1.19 | 0.223 |  |  |  |  | **•** |  |  |  |  |  |  |  |  |
| Map2k3 | -1.79 | 0.009 | -1.60 | 0.046 |  |  |  |  | **•** |  |  |  |  |  |  |  |  |
| Map2k6 | 1.15 | 0.556 | 1.28 | 0.172 |  | **•** |  |  | **•** |  |  |  |  |  |  |  |  |
| Mapk14 | -1.23 | 0.332 | -1.61 | 0.192 |  | **•** |  |  | **•** | **•** |  |  |  |  |  |  |  |
| Mdm2 | -1.10 | 0.669 | -1.36 | 0.281 | **•** |  |  |  |  |  |  |  |  |  |  |  |  |
| Morc3 | 1.13 | 0.624 | 1.67 | 0.056 |  | **•** |  |  |  |  |  |  |  |  |  |  |  |
| Myc | -2.80 | 3X10^-4^ | -3.02 | 0.003 |  | **•** |  |  |  |  |  | **•** |  |  |  |  |  |
| Nbn | -1.35 | 0.223 | -1.24 | 0.297 |  |  |  |  |  |  | **•** |  |  |  |  |  |  |
| Nfkb1 | 1.58 | 0.044 | 3.10 | 0.001 |  |  | **•** |  |  |  |  |  |  |  |  |  |  |
| Nox4 | 2.29 | 0.029 | -1.42 | 0.151 |  |  |  |  |  | **•** |  |  |  |  |  |  |  |
| Pcna | -2.04 | 0.080 | -1.17 | 0.525 |  | **•** |  |  |  |  | **•** |  |  |  |  |  |  |
| Pik3ca | 1.05 | 0.805 | -1.26 | 0.370 |  | **•** |  |  |  |  |  |  |  |  | **•** |  |  |
| Plau | 1.55 | 0.178 | -1.33 | 0.394 |  | **•** |  |  |  |  |  |  |  |  | **•** |  |  |
| Prkcd | 1.36 | 0.040 | 1.60 | 0.028 |  |  |  |  |  | **•** |  |  |  |  |  |  |  |
| Pten | 1.01 | 0.857 | -1.43 | 0.144 |  |  |  |  |  |  |  |  |  |  |  |  | **•** |
| Rb1 | 1.30 | 0.008 | -1.33 | 0.031 | **•** |  | **•** |  |  |  |  |  |  |  |  |  |  |

| (continued) | |  |  |  | **Gene groupings by function** | | | | | | | | | | | | |
| --- | --- | --- | --- | --- | --- | --- | --- | --- | --- | --- | --- | --- | --- | --- | --- | --- | --- |
|  |  |  |  |  | **Senescence pathway** | **Initiators** | | | | | | **Responses** | | | | | |
|  |  |  |  |  |  | **p53/pRb signalling** | **Interferon related** | **Insulin growth factor related** | **MAPK signalling** | **Oxidative stress** | **DNA damage** | **p53 effectors** | **p21 Effectors** | **p16 effectors** | **Cytoskeleton related** | **Cell Adhesion** | **Other (senescence responses)** |
|  |  |  |  |  |  |  |  |  |  |  |  |  |  |  |  |  |  |
|  |  |  |  |  |  |  |  |  |  |  |  |  |  |  |  |  |  |
|  | **15 days of age** | | **25 days of age** | |  |  |  |  |  |  |  |  |  |  |  |  |  |
| **Gene** | **Fold Change** | ***P*** | **Fold Change** | ***P*** |  |  |  |  |  |  |  |  |  |  |  |  |  |
| Rbl1 | -1.35 | 0.201 | 1.11 | 0.911 |  | **•** |  |  |  |  |  |  |  |  |  |  |  |
| Rbl2 | -1.21 | 0.398 | -1.22 | 0.377 | **•** |  |  |  |  |  |  |  |  |  |  |  |  |
| Serpinb2 | 1.73 | 0.153 | 1.01 | 0.860 |  | **•** | **•** |  |  |  |  |  |  |  |  |  |  |
| Serpine1 | -1.49 | 0.361 | ND* | NA |  | **•** |  |  |  |  |  |  |  |  | **•** |  |  |
| Sirt1 | 1.05 | 0.629 | -1.00 | 0.878 |  | **•** |  |  |  |  |  |  |  |  |  |  |  |
| Sod1 | -1.37 | 0.186 | -1.62 | 0.012 |  |  |  |  |  | **•** |  |  |  |  |  |  |  |
| Sod2 | -2.44 | 0.104 | -1.03 | 0.666 |  |  |  |  |  | **•** |  |  |  |  |  |  |  |
| Sparc | 1.91 | 0.002 | 3.34 | 0.014 |  | **•** |  |  |  |  |  |  |  |  | **•** |  |  |
| Tbx2 | 2.61 | 0.035 | 2.90 | 0.010 |  |  |  |  |  |  |  |  |  | **•** |  |  |  |
| Tbx3 | 1.39 | 0.355 | -4.43 | 0.010 |  |  |  |  |  |  |  |  |  | **•** |  |  |  |
| Terf2 | -1.38 | 0.313 | -1.59 | 0.145 |  |  |  |  |  |  | **•** |  |  |  |  |  |  |
| Tert | 1.25 | 0.387 | -1.44 | 0.125 |  |  |  |  |  |  | **•** |  |  |  |  |  |  |
| Tgfb1 | 2.16 | 0.020 | 4.07 | 0.009 |  | **•** |  |  |  |  |  |  |  |  |  | **•** |  |
| Tgfb1i1 | 1.83 | 0.052 | 3.40 | 0.019 |  |  |  |  |  |  |  |  |  |  |  | **•** |  |
| Thbs1 | 2.11 | 0.007 | 1.32 | 0.567 |  |  |  |  |  |  |  |  |  |  | **•** | **•** |  |
| Trp53 | 1.25 | 0.172 | 2.10 | 0.001 | **•** |  |  |  |  |  |  |  |  |  |  |  |  |
| Trp53bp1 | 1.47 | 0.281 | 2.46 | 0.006 |  |  |  |  |  |  | **•** |  |  |  |  |  |  |
| Twist1 | 2.41 | 0.196 | 1.56 | 0.040 | **•** |  |  |  |  |  |  |  |  |  |  |  |  |
| Vim | -1.05 | 0.802 | 2.50 | 0.001 |  |  |  |  |  |  |  |  |  |  | **•** |  |  |
| Gusb | 1.15 | 0.451 | -1.11 | 0.622 | **Control genes** |  |  |  |  |  |  |  |  |  |  |  |  |
| Hprt1 | -1.57 | 0.120 | -1.68 | 0.113 |  |  |  |  |  |  |  |  |  |  |  |  |  |
| Hsp90ab1 | -1.20 | 0.064 | -1.33 | 0.012 |  |  |  |  |  |  |  |  |  |  |  |  |  |
| Gapdh | -1.13 | 0.815 | -1.01 | 0.857 |  |  |  |  |  |  |  |  |  |  |  |  |  |
| Actb | 1.84 | 0.001 | 2.50 | 0.035 |  |  |  |  |  |  |  |  |  |  |  |  |  |

*Not determined. Ct values for Serpine1 were >34 for both *Sbds^P-/R126T^* and *Sbds^P-/+^* at this time point and in subsequent validation sets indicating very low expression levels in this tissue that do not allow for interpretation.
