# Supplementary material for: In Vivo Senescence in the Sbds-Deficient Murine Pancreas: Cell-Type Specific Consequences of Translation Insufficiency
Source: PLoS Genet. 2015 Jun 9;11(6):e1005288. doi: 10.1371/journal.pgen.1005288 (PMC4461263; doi:10.1371/journal.pgen.1005288)
Supplement: S3 Table — Myeloid progenitors were counted following cultivation of equal cell numbers of single organ suspensions of fetal livers of mutant and control embryos at E16.5. N corresponds to the number of embryos analysed for each indicated genotype. (DOCX) [file pgen.1005288.s011.docx]

**Supporting Table 3. Myeloid progenitors of SDS embryos levels improve in the absence of p53**

| Colony counts | | |  |  |  |  |
| --- | --- | --- | --- | --- | --- | --- |
|  | N | CFU-GEMM | CFU-GM | CFU-M | CFU-G | BFU-E |
| *Sbds^+/+^; Trp53^+/-^* | 6 | 9±2 | 31±8 | 25±9 | 44±8 | 19±5 |
| *Sbds^+/+^; Trp53^-/-^* | 5 | 14±7 | 35±5 | 33±6 | 37±11 | 17±6 |
| *Sbds^R126T/+^; Trp53^+/-^* | 10 | 10±4 | 31±14 | 23±8 | 37±13 | 14±11 |
| *Sbds^R126T/+^; Trp53^-/-^* | 5 | 12±7 | 44±20 | 28±12 | 48±26 | 22±11 |
| *Sbds^R126T/R126T^; Trp53^+/-^* | 11 | 3±2 | 15±6 | 11±5 | 29±13 | 8±4 |
| *Sbds^R126T/R126T^; Trp53^-/-^* | 4 | 11±6 | 34±11 | 23±12 | 51±12 | 20±5 |
| Kruskal-Wallis *P*-value |  | 2.9X10^-5^ | 7.6X10^-5^ | 0.00015 | 0.01 | 0.00069 |
|  |  | Wilcoxon Rank Sum Test *P*-value | | | | |
| *Sbds^R126T/R126T^; Trp53^+/-^ vs. controls** | | 6.3X10^-6^ | 1.7X10^-5^ | 2.8X10^-5^ | 0.018 | 0.00053 |
| *Sbds^R126T/R126T^; Trp53^-/-^ vs. controls* | | 0.98 | 0.93 | 0.67 | 0.1 | 0.44 |
| *Sbds^R126T/R126T^; Trp53^+/-^ vs. Trp53^-/-^* | | 0.015 | 0.02 | 0.058 | 0.018 | 0.0049 |

CFU, colony forming unit; GEMM, granulocyte/ erythroid/macrophage /megakaryocyte; GM, granulocyte/macrophage; M, macrophage; G, granulocyte; BFU-E, burst forming unit-erythroid.

**controls* refers to the grouping of *Sbds^+/+^* and *Sbds^R126T/+^* genotypes in the respective *Trp53* genotype background.
