# Supplementary material for: In Vivo Senescence in the Sbds-Deficient Murine Pancreas: Cell-Type Specific Consequences of Translation Insufficiency
Source: PLoS Genet. 2015 Jun 9;11(6):e1005288. doi: 10.1371/journal.pgen.1005288 (PMC4461263; doi:10.1371/journal.pgen.1005288)
Supplement: S4 Table — Sources of all primary and secondary antibodies used in immunohistochemistry and immunoblotting procedures are listed. (DOCX) [file pgen.1005288.s012.docx]

**Supporting Table 4. Antibodies used in this study**

| **Antibody** | **Species** | **Source** |
| --- | --- | --- |
| **Immunohistochemistry** |  |  |
| *Primary antibodies* |  |  |
| Hes1^a^ | Rabbit | Millipore (AB5702) |
| p53* | Rabbit | Novocastra(CM5) |
| Pdx1^a^ | Rabbit | Abcam (AB47267) |
| Prosurfactant Protein C^b^ | Rabbit | Millipore (AB3786) |
| Clara cell 10^b^ | Mouse | Santa Cruz (9772) |
|  |  |  |
| *Secondary antibodies* |  |  |
| ^a^VECTASTAIN® ABC Kit (Rabbit IgG) |  | Vector Labs (PK-4001) |
| ^b^Goat anti-rabbit IgG biotin-SP conjugate |  | Millipore (AP132B) |
|  |  |  |
| **Immunoblotting** |  |  |
| *Primary antibodies* |  |  |
| Sbds | Mouse | In house^†^ |
| Gapdh | Rabbit | Abcam (ab9485) |
| c-Myc | Rabbit | Cell Signaling Technologies (5605) |
| p21^Cip^ | Mouse | BD Pharmingen (556431) |
| p53 | Rabbit | Novocastra (CM5) |
| Smad2 | Rabbit | Cell Signaling Technologies (5339) |
| Phospho-Smad2 | Rabbit | Cell Signaling Technologies (3108) |
| Smad3 | Rabbit | Cell Signaling Technologies (9523) |
| Phospho-Smad3 | Rabbit | Millipore (07-1389) |
| Smad4 | Rabbit | Cell Signaling Technologies (9515) |
| Tgf-β | Rabbit | Cell Signaling Technologies (3711) |
|  |  |  |
| *Secondary antibodies* |  |  |
| Goat anti-rabbit HRP conjugated |  | Bio-Rad (172-1019) |
| Sheep anti-mouse HRP conjugated |  | Amersham (NA931) |
|  |  |  |
|  |  |  |

*p53 immunohistochemistry was completed by the Pathology Core at the Toronto Centre for Phenogenomics.
^†^Monclonal Antibody Facility (S. Arya) at The Hospital for Sick Children, validated in house by R.G.
