# Supplementary material for: In Vivo Senescence in the Sbds-Deficient Murine Pancreas: Cell-Type Specific Consequences of Translation Insufficiency
Source: PLoS Genet. 2015 Jun 9;11(6):e1005288. doi: 10.1371/journal.pgen.1005288 (PMC4461263; doi:10.1371/journal.pgen.1005288)
Supplement: S5 Table — Sequences of all oligonucleotide primers used for the gene expression studies are listed. (DOCX) [file pgen.1005288.s013.docx]

**Supporting Table 5. Oligonucleotides used in this study**

| **Gene of interest** | **Sequence (5'-3')** |
| --- | --- |
| *Cdkn2b (p15^Ink4b^)** | NM_007670 Reference Position 1135 |
| *Cdkn2a (p21^Cip^)* | GCGGTGTCAGAGTCTAGGGGAA |
|  | GCGGAACAGGTCGGACATCAC |
| *E-cadherin* | GAGCGTGCCCCAGTATCGT |
|  | GGCTGCCTTCAGGTTTTCATC |
| *Fn1* | AGCTCATCGGTGATTGTGTC |
|  | CTGCTTGTCAGTGTGTCCTT |
| *Kras* | TGAGAACTGGGGAGGGCTTTCTT |
|  | ACCAGGACCATAGGCACATCTTCA |
| *Myc*^†^ | CCTAGTGCTGCATGAGGAGACA |
|  | CCTCATCTTCTTGCTCTTCTTCAGA |
| *Tgfb1* | ACAATTCCTGGCGTTACCTT |
|  | TGGAGTTTGTTATCTTTGCTGTCA |
| *TgfbrI* | GCGAACAGAAGTTAAGGCCA |
|  | TCCATTGGCATACCAGCATT |
| *TgfbrII* | AGGACCATCCATCCACTGAAA |
|  | GACAGTCTCACATCGCAAAAC |
| *TgfbrIII* | CCCAGATGGTGTGGTTTACTA |
|  | GGCAGTTTCTCCTTCATCTGT |
| *Trp53* | TCAAGATCCGCGGGCGTAAAC |
|  | CAAGGCTTGGAAGGCTCTAGGC |
| *Actb* | CTGCTCTGGCTCCTAGCACCA |
|  | CAGCTCAGTAACAGTCCGCCTAGAA |
| *Gapdh* | TCACCACCATGGAGAAGGC |
|  | GCTAAGCAGTTCGTGGTGCA |
| *Gusb* | CCGATTATCCAGAGCGAGTATG |
|  | CTCAGCGGTGACTGGTTCG |
| *Tbp* | CAAACCCAGAATTGTTCTCCTT |
|  | ATGTGGTCTTCCTGAATCCCT |

*Purchased from QIAGEN (p15^Ink4b^ Cat. no. PPM02910E-200)

^†^Strom, A. *et al.* Unique mechanisms of growth regulation and tumor suppression upon Apc inactivation in the pancreas. *Development* 134, 2719-2725 (2007).
